# IMPROVE-DiCE, a 2-Part, Open-Label, Phase 2a Trial Evaluating the Safety and Effectiveness of Ninerafaxstat in Patients With Cardiometabolic Syndromes

**DOI:** 10.1161/CIRCULATIONAHA.125.074041

**Published:** 2025-12-18

**Authors:** Moritz J. Hundertmark, Sarah M. Birkhoelzer, Clara Portwood, Adrienne G. Siu, Violet Matthews, Andrew J. Lewis, James Grist, Ferenc Mózes, John A. Henry, Jai Patel, Paul Chamberlin, Rizwan Sarwar, Arash Yavari, Hakim-Moulay Dehbi, Prashant Rao, Xu Shi, Shuning Zheng, Jeremy M. Robbins, Robert E. Gerszten, Michael P. Frenneaux, Ladislav Valkovič, Jack J.J.J. Miller, Stefan Neubauer, Damian J. Tyler, Oliver J. Rider

**Affiliations:** 1 Oxford Centre for Clinical Magnetic Resonance Research, Division of Cardiovascular Medicine, Radcliffe Department of Medicine, University of Oxford, John Radcliffe Hospital, Oxford, United Kingdom (M.J.H., S.M.B., C.P., A.G.S., A.J.L., J.G., F.M., J.A.H., L.V., S.N., D.J.T., O.J.R.).; 2 Department of Cardiology, Inselspital, Bern University Hospital, University of Bern, Bern, Switzerland (M.J.H.).; 3 Department of Physiology, Anatomy and Genetics, Medical Sciences Division, Oxford, United Kingdom (A.G.S., J.G., D.J.T.).; 4 Oxford University Hospitals NHS Foundation Trust, Oxford, United Kingdom (V.M., R.S.).; 5 Department of Radiology, Oxford University Hospitals NHS Foundations Trust, Oxford, United Kingdom (J.G.).; 6 Institute of Cancer and Genomic Sciences, University of Birmingham, Birmingham, United Kingdom (J.G.).; 7 Imbria Pharmaceuticals, Boston, MA (J.P., P.C., A.Y.).; 8 Experimental Therapeutics, Radcliffe Department of Medicine, University of Oxford, Oxford, United Kingdom (R.S., A.Y.).; 9 Comprehensive Clinical Trials Unit, University College London, London, United Kingdom (H.-M.D.).; 10 Division of Cardiovascular Medicine, Beth Israel Deaconess Medical Center, Boston, MA (P.R., X.S., S.Z., J.M.R., R.E.G.).; 11 CardioVascular Institute, Beth Israel Deaconess Medical Center, Boston, MA (P.R., X.S., S.Z., J.M.R., R.E.G.).; 12 Imperial College London (M.P.F.).; 13 Department of Imaging Methods, Institute of Measurement Science, Slovak Academy of Sciences, Bratislava, Slovakia (L.V.).; 14 Department of Clinical Medicine, Aarhus University, Aarhus, Denmark (J.J.J.J.M.).

**Keywords:** cardiac energy metabolism, diabetic cardiomyopathy, HFpEF, hyperpolarized MR, magnetic resonance imaging, obesity

## Abstract

**BACKGROUND:**

We report IMPROVE-DiCE (Improve Diabetic Cardiac Energetics), a 2-part open-label, phase 2a trial evaluating the safety and effectiveness of ninerafaxstat, a novel therapeutic designed to enhance cardiac energetics. Between May and September 2021, part 1 enrolled patients with type 2 diabetes and obesity without heart failure with preserved ejection fraction (HFpEF). Between January 2023 and June 2024, part 2 enrolled patients with type 2 diabetes, obesity, and HFpEF.

**METHODS:**

Forty-two participants received 200 mg ninerafaxstat twice daily (part 1, n=21, 43% women, 72±0.5 years of age, 4–8 weeks; part 2, n=21, 29% women, 71±6 years of age, 12 weeks). Myocardial energetics (phosphocreatine-to-ATP ratio [PCr/ATP], primary outcome) and function (rest and dobutamine stress) were assessed before and after treatment using magnetic resonance imaging, ^31^P- and ^1^H magnetic resonance spectroscopy. In part 1, hyperpolarized [1-^13^C]pyruvate magnetic resonance spectroscopy to assess in vivo pyruvate dehydrogenase flux (n=9) and plasma metabolomics and proteomics were also performed.

**RESULTS:**

In part 1, in patients with diabetes and obesity but without HFpEF, the heart was characterized by impaired pyruvate dehydrogenase flux, reduced PCr/ATP, triglyceride deposition, and diastolic impairment. Treatment with ninerafaxstat was associated with improved PCr/ATP (+0.39±0.49 [95% CI, 0.16–0.62]; Cohen’s *d*, 0.79; *P*=0.002) and lower myocardial triglyceride (by 34%, *P*=0.03). In part 2, in patients with diabetes, obesity, and symptomatic HFpEF, the heart was characterized by reduced PCr/ATP, diastolic impairment, and failure of systolic augmentation to exercise. Consistently, treatment with ninerafaxstat was associated with improvement in PCr/ATP (+0.15±0.25 [95% CI, 0.03–0.26]; Cohen’s *d*, 0.60; *P*=0.02), improved systolic augmentation to exercise (+1.4 L/min, *P*=0.04), improved exercise capacity (6-minute walk distance +16 m, *P*=0.02), and improved New York Heart Association class symptom burden.

**CONCLUSIONS:**

These mechanistic phase 2a study results show that ninerafaxstat is safely tolerated and improves myocardial energetics in participants with obesity and diabetes without or with clinically manifest HFpEF.

**REGISTRATION:**

URL: https://www.clinicaltrials.gov; Unique identifier: NCT04826159.

**Clinical Perspective**

**What Is New?**

- Ninerafaxstat combines a trimetazidine analogue, inhibiting mitochondrial 3-ketoacyl-coenzyme A thiolase, a key enzyme in fatty acid β-oxidation, with nicotinamide adenine dinucleotide, critical for mitochondrial redox balance, metabolic flexibility, and oxidative stress resistance, improving both myocardial substrate availability and oxidative capacity.
- This study combined multinuclear magnetic resonance spectroscopy, in vivo magnetic resonance hyperpolarization, and imaging parameters with functional and clinical assessments, showcasing that metabolic imaging can demonstrate improvement in substrate use and oxidation, which, in this study, was linked to improved exercise performance and symptoms in participants with cardiometabolic heart failure with preserved ejection fraction.

**What Are the Clinical Implications?**

- Ninerafaxstat was well tolerated and associated with improved cardiac energetic state, cardiac function, and patient-reported outcomes in individuals with metabolic heart failure with preserved ejection fraction .
- This study shows that modulation of myocardial substrate use may offer a new treatment strategy in patients with cardiometabolic heart failure with preserved ejection fraction, a population with limited treatment options.
- If confirmed in larger trials, altering myocardial metabolism could shift the treatment paradigm for heart failure with preserved ejection fraction, focusing on the treatment of comorbidities to modulate substrate use and targeting underlying myocardial energy deficits with cardiac mitotropes.

**Editorial, see p 564**

Type 2 diabetes (T2D) and obesity are accompanied by significant myocardial metabolic alterations and both structural and functional cardiac changes, including left ventricular (LV) hypertrophy, diastolic, and systolic dysfunction.^1^ Even in the absence of traditional risk factors, such as coronary artery disease or hypertension, this represents a pre–heart failure (HF) state that may then progress to clinically overt HF. Although they are linked to incidence of various HF phenotypes, the presence of T2D and obesity substantially increases the risk of developing HF with preserved ejection fraction (HFpEF).^2–4^ Given this link to HFpEF and the continual global increase in T2D, the prevalence of diabetes-related HFpEF is projected to further increase.^5^ As a result, there is a substantial unmet medical need for novel therapeutics treating the spectrum of diabetes-related cardiac dysfunction from subclinical through to established HFpEF.^6^ One such novel therapeutic intervention is altering myocardial metabolism.

The energy requirements of the heart are unmitigated, and it is the highest energy consumer (ATP per gram of tissue) in the body, with a complete turnover of its ATP pool approximately every 10 s.^7^ Therefore, any mismatch between ATP provision and demand leads to an energetic deficit and cardiac dysfunction, exacerbated further in the context of increased workload.^8^ Under physiological conditions, the healthy heart is metabolically flexible and able to dynamically use a range of carbon-based substrates to generate ATP. Free fatty acids (FFAs) and glucose, as well as contributions from lactate, ketone bodies, and several amino acids, ensure close coupling of mitochondrial ATP production with the rate of ATP use across a range of states, such as feeding, fasting, and exercise.^9^

Conversely, obesity and diabetes increase levels of circulating FFAs and glucose^10^ with an overreliance on FFAs to generate ATP in the context of progressive insulin resistance.^11^ This likely results in loss of metabolic flexibility,^12^ lipotoxicity, and inhibition of pyruvate dehydrogenase (PDH), resulting in uncoupling of glycolysis from glucose oxidation.^5^ Over time, this metabolic remodeling increases myocardial oxygen consumption, oxidative stress, and inflammation but also reduces cardiac efficiency.^13^ Consequently, T2D and obesity are both linked to a lower phosphocreatine-to-ATP ratio (PCr/ATP, a sensitive marker of energetic status), evident early in the disease process.^14,15^ Hence, the insulin-resistant heart displays reduced PDH flux,^16^ impaired myocardial energetics,^17^ cardiac steatosis,^18^ LV hypertrophy, and LV diastolic or systolic dysfunction.^19^ This shares many of the phenotypic features of HFpEF, in which LV hypertrophy, diastolic impairment, lipotoxicity, and reduced PCr/ATP are also seen.^20,21^

Ninerafaxstat is a novel therapeutic designed to shift myocardial substrate use in favor of glucose oxidation through partial fatty acid oxidation (FAO) inhibition, thus increasing PDH activity, and use of a precursor in the synthesis of nicotinamide adenine dinucleotide (NAD^+^) to enhance the cellular NAD^+^ pool. These effects aim to restore substrate flexibility and improve cardiac energetics, metabolism, and function. Ninerafaxstat undergoes rapid hydrolysis during enteral absorption and in plasma to liberate IMB-1028814, a novel structural analogue of trimetazidine (TMZ; with a portion undergoing further metabolism to TMZ) and nicotinic acid. In rodents, ninerafaxstat markedly increases myocardial glucose uptake and exerts protective effects in preclinical models of ischemia/reperfusion injury and pressure overload.^22^ Recently, ninerafaxstat showed improvements in exercise tolerance in patients with nonobstructive hypertrophic cardiomyopathy,^23^ a condition driven by LV hypertrophy, diastolic dysfunction, and impaired cardiac energy metabolism, equally prevalent in T2D, obesity, and HFpEF.

In this article, we present results of a phase 2a clinical trial that sought to examine the effects of ninerafaxstat on myocardial function, energetics, and metabolism using cardiovascular magnetic resonance (MR), echocardiography, and trinuclear (^31^P, ^1^H, and ^13^C) MR spectroscopy (MRS). Part 1 evaluated participants with T2D and obesity who are at risk of HFpEF, and part 2 evaluated participants with T2D, obesity, and HFpEF.

## METHODS

IMPROVE-DiCE (Improve Diabetic Cardiac Energetics) was a 2-part, open-label, phase 2a trial evaluating the safety and effectiveness of ninerafaxstat. Part 1 enrolled participants with T2D and obesity who were at risk of HFpEF and part 2 participants with T2D, obesity, and HFpEF.

The trial was registered (EudraCT No. 2020-003280-26; URL: https://clinicaltrials.gov; Unique identifier: NCT04826159) and sponsored by Imbria Pharmaceuticals, Inc. Ethics approval for the study was granted by the Medicines and Healthcare Products Regulatory Agency and National Research Ethics Service (research ethics committee reference 20/LO/1120). The trials were conducted in accordance with the principles of the Declaration of Helsinki and the European Union Clinical Trials Directive. All participants provided written informed consent before any investigations. Study visits took place at the Oxford Centre for Clinical Magnetic Resonance at the John Radcliffe Hospital, Oxford, United Kingdom. Data will be available upon request.

### Inclusion and Exclusion Criteria

#### Part 1: Participants With Obesity and T2D

**Figure 1.**
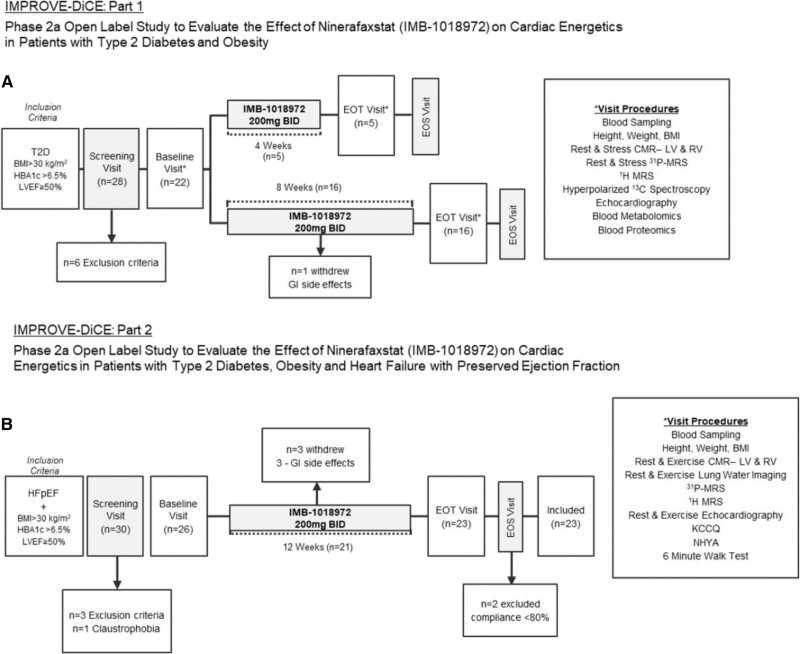
**Flowchart of study procedures of the 2 studies. A**, The participant flow examining participants with type 2 diabetes (T2D) and obesity being treated for 4 or 8 weeks. **B**, The second part of our 2-fold study design, involving participants with T2D, obesity, and heart failure with preserved ejection fraction (HFpEF), all being treated for 12 weeks.

Inclusion criteria were as follows: 18 to 75 years of age with T2D (hemoglobin A1c [HbA_1c_] ≥6.5%), a body mass index (BMI) ≥30 kg/m^2^, and preserved LV ejection fraction (LVEF) ≥50%). Exclusion criteria were as follows: insulin or sodium-glucose cotransporter 2 inhibitor (SGLT2i) therapy, more than moderate renal impairment (estimated glomerular filtration rate <60 mL/min per 1.73 m^2^), HF, any change in oral antidiabetic therapy in the past 3 months, and contraindications to MR scanning. A detailed list of inclusion and exclusion criteria can be found in Table S1. The first 5 participants received ninerafaxstat (200 mg BID) for 4 weeks, with the subsequent 16 participants receiving ninerafaxstat (200 mg BID) for 8 weeks. All analyses for this article are from the combined 4- and 8-week treatments. A flowchart of the study procedures is shown in Figure 1A. A full medication list is shown in Table S2.

#### Part 2: Participants With Obesity, T2D, and HFpEF

Inclusion criteria were as follows: 18 to 80 years of age with T2D (HbA_1c_ ≥6.5%), a BMI ≥30 kg/m^2^, LVEF ≥50%, clinically stable symptomatic HF (New York Heart Association (NYHA) functional class II to III), and diagnosis of HFpEF by the Heart Failure Association pretest assessment, echocardiography, functional, final etiology score algorithm^24^ (≥5 points) or heavy (BMI, hypertensive, atrial fibrillation, pulmonary hypertension, elder) score^25^ of ≥6 points. SGLT2i use was permitted in part 2, provided there was stable dosing for ≥3 months at entry. Exclusion criteria were as follows: more than moderate renal impairment (glomerular filtration rate <60 mL/min per 1.73 m^2^), change in oral antidiabetic therapy in the past 3 months, and any contraindications to MR scanning. A detailed list of inclusion and exclusion criteria can be found in the Data Supplement. A flow chart of the study procedures is shown in Figure 1B. All participants received ninerafaxstat (200 mg BID) for 12 weeks. A full medication list is shown in Table S3.

### Trial Oversight

Medpace coordinated and monitored the study, including safety monitoring and data management. A web-based electronic case record form was used. Adverse event (AE) reporting was performed by the study team and independently reviewed by Medpace. Initiation, routine monitoring, and closeout visits were performed on site.

### Outcome Measures

The primary outcome for both studies was change from baseline to end of treatment in myocardial PCr/ATP by ^31^P-MRS. Secondary outcomes included changes from baseline to end of treatment in cardiac systolic and diastolic function, systolic augmentation during stress (dobutamine part 1, exercise part 2), and an evaluation of the safety and tolerability of repeat oral doses. Additionally, we sought to investigate the impact of ninerafaxstat on myocardial PDH flux inferred by the [1-^13^C]bicarbonate–to–[1-^13^C]pyruvate ratio measured by hyperpolarized [1-^13^C]pyruvate MRS in a subset of 9 participants in part 1.

Exploratory end points included changes in myocardial steatosis; myocardial enzymatic fluxes reflecting the metabolic fate of pyruvate, including the lactate dehydrogenase (LDH) and alanine aminotransferase reactions, measured by hyperpolarized [1-^13^C]pyruvate MRS (part 1 only); systemic insulin sensitivity (homeostasis model assessment); lipid profile and circulating NT-proBNP (N-terminal pro-B-type natriuretic peptide) and high-sensitivity cardiac troponin; and readouts of plasma proteomic and metabolomic markers (part 1 only).

### Anthropometric and Biochemical Assessment

Height, weight, and blood pressure were recorded at baseline and after treatment. After overnight fasting, participants’ venous blood samples were drawn, and biomarkers were analyzed according to standardized protocols. Fasting insulin resistance was represented by homeostasis model assessment of insulin resistance (glucose [millimoles per liter]×insulin [picomoles per liter]/135).

### Cardiac Magnetic Resonance Imaging and Spectroscopy

A brief overview of techniques is described below, with additional details provided in the Supplemental Material.

### MR Imaging

Using ECG gated, balanced, steady-state, free precession cine imaging, long- and short-axis images of the left ventricle were acquired. In part 2, a real-time, highly accelerated, compressed sensing, free-breathing cine imaging sequence was used to acquire a short-axis stack during exercise (≈60 s). Image analysis was performed offline in accordance with Society for Cardiovascular Magnetic Resonance guidelines,^26^ using cvi42 postprocessing software (version 5.10.1, Circle Cardiovascular Imaging Inc, Calgary, AB, Canada).

### ^31^P-MRS to Assess Myocardial Energetics

^31^P-MRS was performed on a 3-Tesla MR scanner (Prisma; Siemens Healthineers, Erlangen, Germany). In brief, participants were positioned prone over the center of a 3-element dual-tuned ^1^H/^31^P surface coil (Siemens Medical, Erlangen, Germany) in the MR isocenter. A nongated 3D, acquisition-weighted, ultrashort echo time chemical shift imaging sequence was used, with saturation bands placed over the liver and skeletal muscle, as previously described.^27^ All spectra were analyzed using a semiautomated fitting of data using the Oxford Spectroscopy Analysis Toolbox (OXSA) toolbox,^28^ a MATLAB implementation of the advanced method for accurate and robust estimation of spectra fitting routine performed by experienced operators (M.J.H. and L.V., >5 years of experience). Further details concerning data analysis are provided in the Supplemental Material.

### Hyperpolarized [1-13C]pyruvate Spectroscopy

A SpinLab system (GE Healthcare, Chicago, IL) was used for the process of dynamic nuclear polarization, as described previously.^16^ Intravenous injection of the hyperpolarized pyruvate was undertaken at a dose of 0.4 mL/kg and at a rate of 5 mL/s via a power injector (MEDRAD, Bayer).^16^ Further details regarding spectral analysis are provided in the Supplemental Material.

### ^1^H-MRS to Assess Myocardial Steatosis

All ^1^H-MR assessments were also performed using 3.0-Tesla MR as previously described.^29,30^ In brief, end-expiration, ECG-triggered spectra were obtained from the midinterventricular septum, avoiding vascular structures. Spectra were acquired with and without water suppression to measure myocardial triglyceride content (MTGC). Spectra were analyzed using MATLAB and the advanced method for accurate and robust estimation of spectra algorithm within the Oxford Spectroscopy Analysis Toolbox. MTGC was calculated as a percentage (signal amplitude of lipid/signal amplitude of water)×00.

### Hepatic Fat

Hepatic fat was measured using the Dixon 3-point method to derive a fat fraction. The analysis was performed with MicroDicom Viewer 2024.2 (64 bit). For each participant, MR imaging proton density fat fraction was derived by drawing 3 regions of interest at the level of the main portal vein in a homogenous part of the liver.

### Stress Myocardial Measurements

^31^P-MRS and long-axis cine images were repeated at stress to elicit energetic and contractile responses to increased workload as well as exclude any inducible regional wall motion abnormalities. In part 1, dobutamine was infused via a peripheral venous cannula, as necessary (15–40 μg/kg/min) to achieve a target heart rate of 65% age maximum (calculated as 220−age). In part 2, supine exercise (fixed workload of 30 W for 6 minutes) was performed using a cardiovascular MR–compatible ergometer (Ergospect GmbH, Innsbruck, Austria).

### Lung Water Imaging

To quantify dynamic changes in lung water, a custom proton density lung mapping sequence was used as previously described and validated.^20^ This used a constant low-flip-angle, multiecho ultrashort echo time pulse sequence with a golden-angle radial-out sampling scheme and out-of-slice and fat saturation schemes. With the low flip angle used (5°) and the ability of ultrashort echo time sequences to adequately resolve lung parenchyma, scan parameters were chosen to ensure a linear response of signal intensity to proton density within the lung in its physiological range. A modified radial post hoc reconstruction algorithm was applied to ensure that residual exercise-induced motion artefacts were aliased outside the lung field of view and reconstruct images from the same phase of the cardiac cycle.

### Plasma Metabolomic and Proteomic Analyses

Metabolite profiling was performed using standard liquid chromatography-tandem mass spectrometry. Water-soluble metabolites were profiled in positive ionization mode using an liquid chromatography-tandem mass spectrometry system composed of a Nexera X2 ultra-high-performance liquid chromatograph (Shimadzu Corp, Marlborough, MA) coupled to a Q Exactive mass spectrometer (Thermo Fisher Scientific, Waltham, MA) and equipped with a hydrophilic interaction liquid chromatography column (Atlantis HILIC; Waters, Milford, MA), as previously described.^31^ Proteomic profiling was performed using the Olink 96 cardiovascular panel (Olink Proteomics AB, Uppsala, Sweden) according to manufacturer instructions using separate aliquots. The proximity extension assay technology used for the Olink protocol has been previously described.^32^ Detailed metabolite and protein profiling methods are available in the Supplemental Methods.

### Echocardiography

Echocardiography was performed on a Vivid i system (GE Healthcare, Boston, MA) using a standardized protocol including parasternal long and short axis views as well as apical 2-, 3-, and 4-chamber views. Color Doppler assessments were performed to exclude significant valvular heart disease. Pulsed-wave Doppler assessment of mitral valve inflow was used to calculate E wave (early filling) and an A wave (atrial contraction). Tissue Doppler velocities were measured at the medial and lateral mitral valve annulus to determine E/e′. Continuous-wave Doppler was used to assess tricuspid regurgitation velocity for estimation of systolic pulmonary artery pressure. This was repeated during the 30-W exercise in part 2.

### Statistics

We powered our study assuming a standardized Hedge’s $g^{*}$ effect size of $g^{*}=0.2$, with β=0.8 and α=0.05 on the basis of previous studies with metabolic modulators in our center^33^ that assessed cardiac energetics through ^31^P-MRS (PCr/ATP) or noninvasive assessment of cardiac metabolism using hyperpolarized [1- ^13^C]pyruvate MRS. We therefore included a minimum of 20 participants at 200 mg of ninerafaxstat BID for ^31^P-MRS and hyperpolarized [1- ^13^C]pyruvate MRS data from a minimum of 8 participants. The problem of multiple comparisons was addressed by using the Benjamini-Hochberg correction; a false discovery rate <5% was considered statistically significant. Statistical analyses were performed using commercial software (SPSS 24 and GraphPad Prism 9). All data are presented as median (interquartile range), at which the interquartile range is the 25th percentile, 75th percentile, or mean (SD) unless otherwise stated. Normality was assessed using the Shapiro-Wilk test. Depending on the results, statistical significance was determined using either the paired *t* test or the Wilcoxon signed rank test. Pearson’s or Spearman correlation coefficients and linear regression were used when indicated by normality testing. Values of *P*<0.05 were considered statistically significant. No imputation for missing data was performed.

## RESULTS

### Enrollment and Study Duration

For part 1, participant recruitment took place between May and September 2021. All follow-up visits were completed by December 2021. A total of 28 participants were screened for eligibility, 22 were enrolled. One participant withdrew after 2 days of dosing; another participant did not complete the MR examination at the end-of-trial visit because of claustrophobia. For part 2, participant recruitment took place between January 2023 and June 2024. All follow-up visits were completed by August 2024. A total of 56 participants were screened for eligibility; 26 were enrolled. Three participants withdrew from the study because of AEs. The end-of-treatment visit was attended by 23 participants. Two participants were subsequently excluded because of medication compliance <80%, leaving 21 included data sets.

### Population Characteristics

Baseline characteristics for both studies are shown in the Table. In part 1, the mean age was 71 years, 43% were women, and 100% were White. Mean weight was 97 kg, mean BMI was 33.6 kg/m^2^, and median glycated HbA1c was 7.0%. Ninety percent were taking oral antidiabetic medication. Most participants enrolled were on a statin (81%) and renin-angiotensin-aldosterone system blockade (57%). In study 2, the mean age was 71±6 years, 29% were women, and 100% were White. Mean weight was 104 kg, and mean BMI was 35.2 kg/m^2^. HFpEF was confirmed with a group mean NT-proBNP of 999 ng/mL; median Heart Failure Association pretest assessment, echocardiography, functional, final etiology score of 6; heavy (BMI, hypertensive, atrial fibrillation, pulmonary hypertension, elder) score of 8; and NYHA class of 2. Mean HbA_1_c was 6.4%. Most participants enrolled were on a statin (90%), renin-angiotensin-aldosterone system blockade (81%), or loop diuretic (67%).

**Table.**
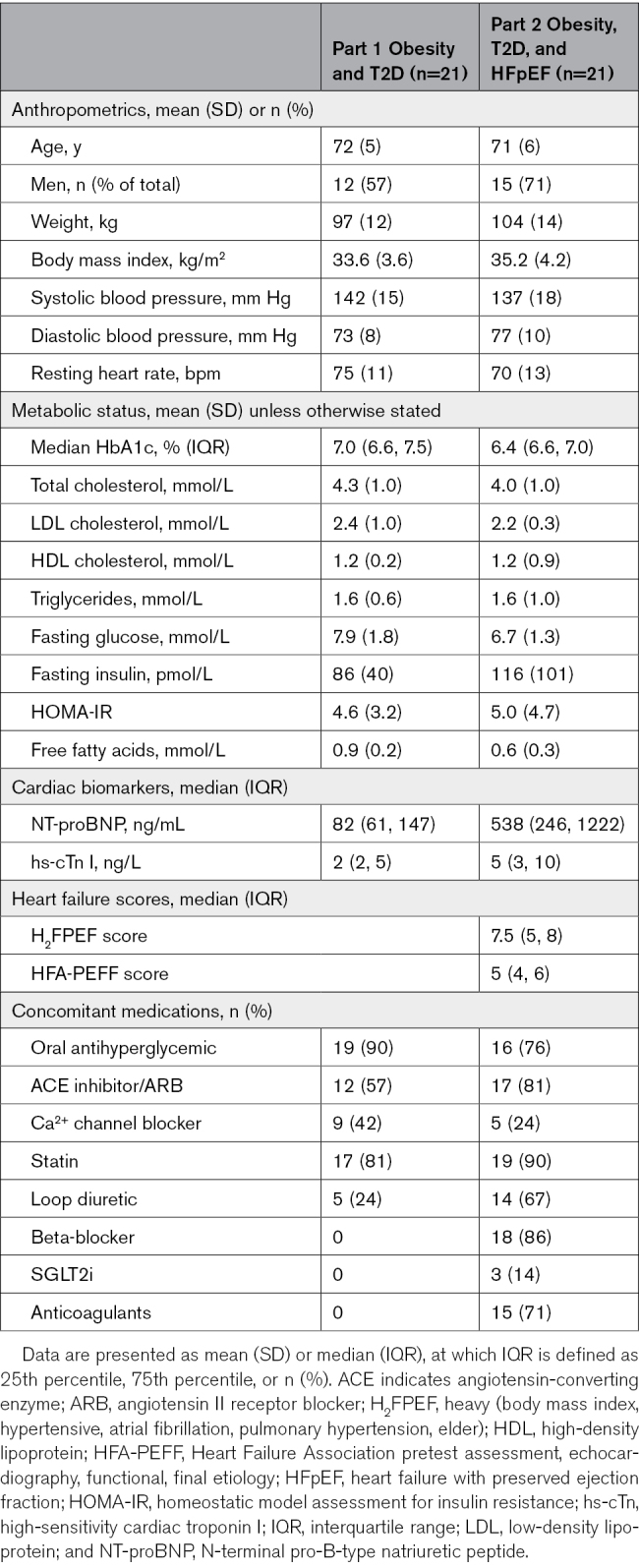
Participant Characteristics at Baseline for IMPROVE-DiCE

### Body Composition and Biomarkers

After treatment, in study 1, there was a significant reduction in overall body weight (by 0.8±1.4 kg, *P*=0.04) and low-density lipoprotein cholesterol (by 0.2±0.4 mmol/L, *P*=0.03). There was a small but clinically insignificant rise in urea (0.3±0.5 mmol/L, *P*=0.01) and creatine kinase (21±41 mmol/L, *P*=0.03). Fasting glucose, FFAs, HbA1c, insulin, and triglycerides were all unchanged, along with other measures of renal and hepatic function (Table S4). In study 2, treatment with ninerafaxstat was again associated with a small reduction in BMI (0.4±0.8 kg/m^2^, *P*=0.04). There was no significant change in systolic or diastolic blood pressure, renal or hepatic function, troponin, or NT-proBNP. Fasting glucose, insulin, lipid profile, FFAs, and ketones were again all unchanged (Table S5).

### Myocardial Metabolism

### ^31^P- and ^1^H-MRS

**Figure 2.**
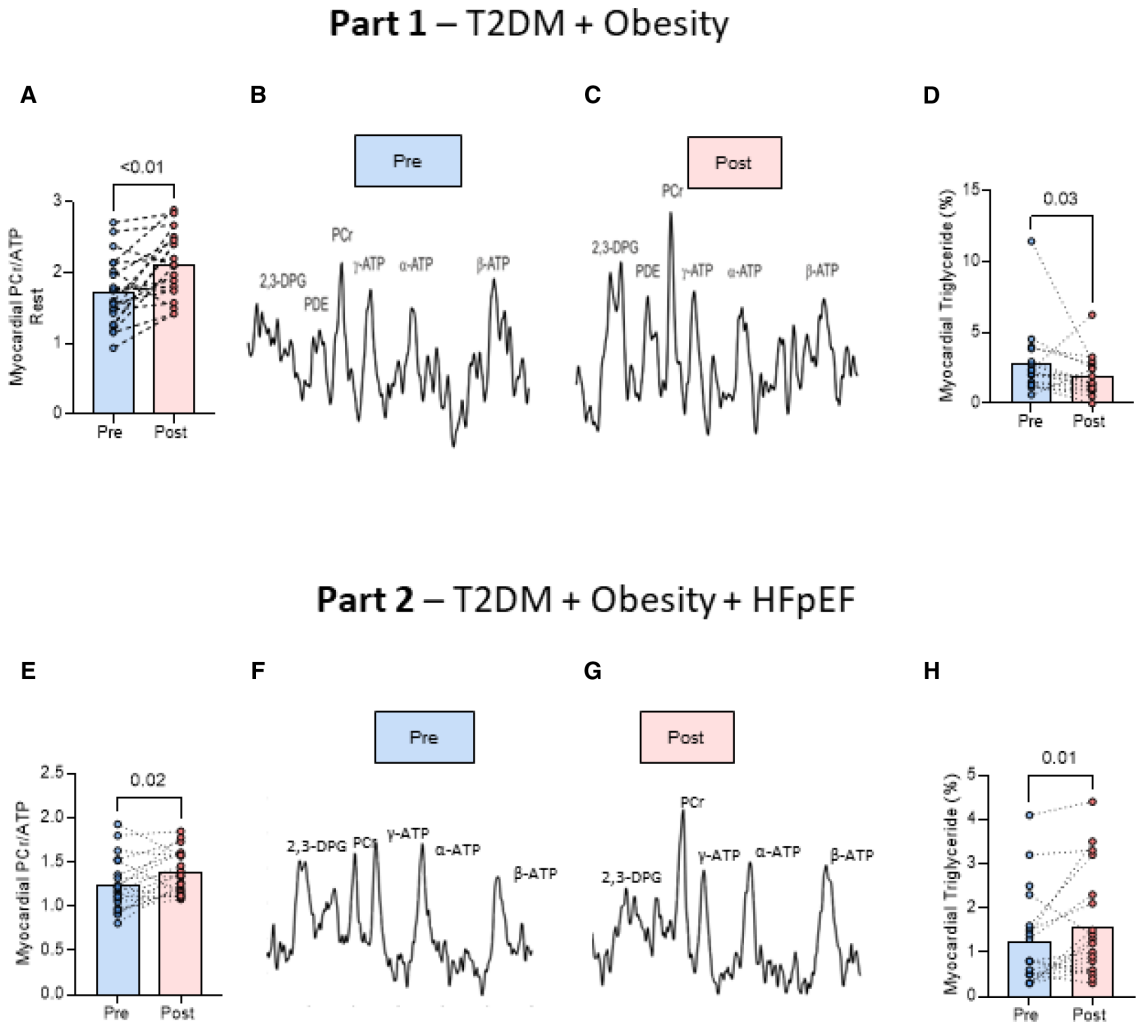
**The effect of ninerafaxstat on metabolic readouts.** Part 1 involved participants with type 2 diabetes (T2D) and obesity but without heart failure with preserved ejection fraction (HFpEF). **A** through **C**, magnetic resonance spectroscopy results. **A**, The increase in resting myocardial phosphocreatine-to-ATP ratio (PCr/ATP; primary outcome). **B** and **C**, A before and after spectrum, respectively. **D**, The drop in myocardial triglyceride content after treatment. Part 2 involved participants with T2D and obesity and HFpEF. **E**, The increase in resting myocardial PCr/ATP (primary outcome). **F** and **G**, A before and after spectrum, respectively. **H**, The increase in myocardial triglyceride content after treatment.

Figure 2 shows the effect of ninerafaxstat on metabolic readouts, including ^31^P-MRS and ^1^H-MRS data. In study 1, after ninerafaxstat treatment, myocardial energetics were improved at rest, with a 32% increase in PCr/ATP (1.72±0.48 versus 2.12±0.48, *P*<0.01; Figure 2A). Example spectra in one participant are shown in Figure 2B and 2C. No change in stress PCr/ATP during dobutamine infusion was seen (PCr/ATP rest 1.8±0.25 versus stress 1.8±0.33, *P*>0.9). After treatment with ninerafaxstat, MTGC was significantly reduced by 34% (Figure 2D). In a similar fashion, in part 2, after treatment with ninerafaxstat, resting myocardial energetics were improved, with an increase in PCr/ATP (Figure 2E). Examples of the improvement in rest PCr/ATP in 1 participant are shown in Figure 2F and 2G. In contrast, treatment with ninerafaxstat was associated with an increase in MTGC (Figure 2H).

### Carbon-13 Magnetic Resonance Spectroscopy

Figure S1 shows the hyperpolarized carbon-13 magnetic resonance spectroscopy data for part 1. The [^13^C]bicarbonate–to–[1-^13^C]pyruvate ratio, a measure of enzymatic PDH flux, was lower than literature values in controls^16^ and related to insulin resistance (homeostasis model assessment of insulin resistance, *r* −0.67, *P*=0.04; Figure S1A) and to resting PCr/ATP (Figure S1B). As a marker of balance between glycolytic and oxidative carbohydrate metabolism, the ratio of [^13^C]bicarbonate and [1-^13^C]lactate signals showed a significant reduction in relative carbohydrate oxidation in T2D and was related to PCr/ATP (Figure S1C), suggesting that higher levels of glucose oxidation are associated with better energetics. After ninerafaxstat treatment, the [1-^13^C]bicarbonate/pyruvate ratio was, in absolute terms, increased in 7 of 9 patients and overall by 20% (Figure S1D) but did not reach nominal significance. The [1-^13^C]alanine/pyruvate ratio was reduced by 20% after treatment (*P*=0.05; Figure S1E). Both the [1-^13^C]lactate/pyruvate (Figure S1F) and [^13^C] bicarbonate/lactate signal ratio, although increased, remained unchanged with treatment. In addition, the change in [1-^13^C]bicarbonate/pyruvate ratio was related to the change in insulin and glucose during the study (Figure S1G; S1H). However, the change in [^13^C] bicarbonate/lactate was related to the change in insulin (Figure S1I).

### Metabolomics and Proteomics for Part 1

Figure S2) shows proteomic and metabolomic changes in study 1. Plasma metabolomics before and after treatment showed a significant increase in levels of 1-methylnicotinamide (false discovery rate–adjusted *P*<0.05), N1-methyl-2-pyridone-5-carboxamide, and niacinamide, which are downstream metabolites of niacin, an active moiety of ninerafaxstat (Figure S2A). Proteomic analyses of the same sampled revealed a significant upregulation of CTSL1 (cathepsin L1; false discovery rate–adjusted *P*<0.05), whereas a set of proteins relating to cardiac remodeling (lectin-like oxidized low-density lipoprotein receptor 1), inflammation (matrix metalloproteinase 9), and diastolic dysfunction (fatty acid binding protein 4) showed a trend toward downregulation after ninerafaxstat treatment (Figure S2B).

### Myocardial Function

#### Left Ventricle

**Figure 3.**
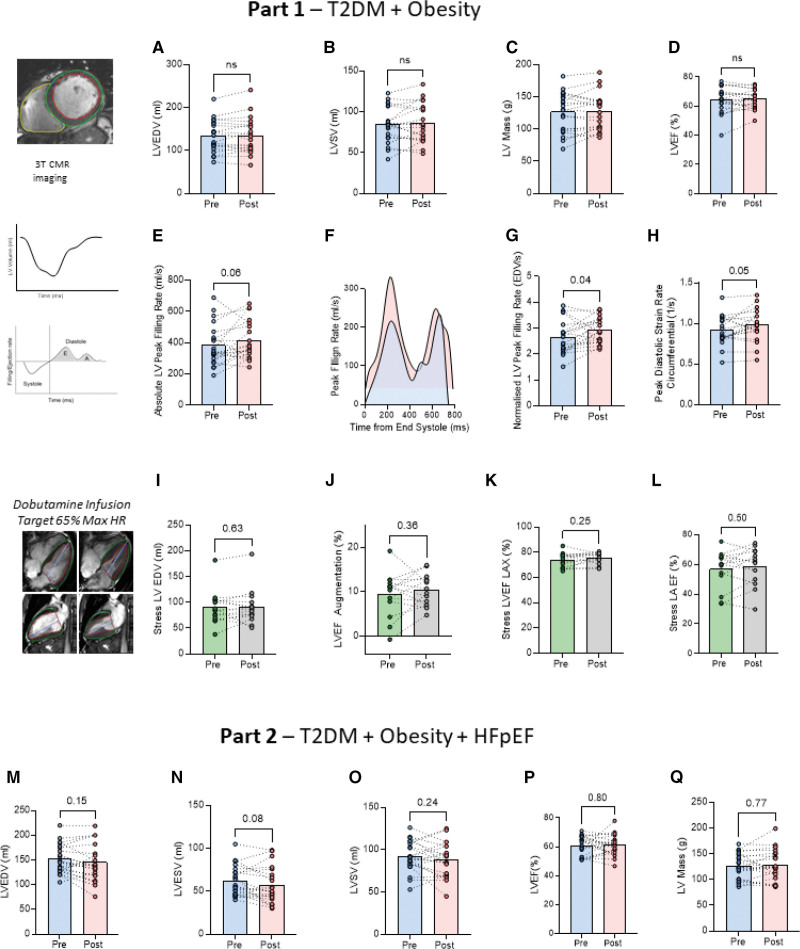
**The effect of ninerafaxstat on cardiac morphology and function.** Part 1 involved participants with type 2 diabetes (T2D) and obesity without heart failure with preserved ejection fraction (HFpEF). **A** through **D**, Left ventricular (LV) end-diastolic volume (EDV), left ventricular stroke volume (LVSV), LV mass, and left ventricular ejection fraction (LVEF). **E** through **H**, Diastolic filling rates and strain. **I** through **K**, Right ventricular (RV) EDV, end-systolic volume (ESV), right ventricular SV (RVSV), and right ventricular ejection fraction (RVEF). **l** shows left atrial (LA) size. Part 2 involved participants with T2D and obesity and HFpEF. **M** through **P**, LV size and systolic function. **Q**, LV mass.

In study 1, whereas short-term treatment with ninerafaxstat was not associated with changes in LV end-diastolic volume, stroke volume (SV), mass, or LVEF (Figure 3A through 3D), there was improvement in diastolic function, with LV filling rates (Figure 3E through 3G) and peak diastolic strain rate (Figure 3H) being higher after treatment. Ninerafaxstat did not change LV size or systolic function during dobutamine stress (Figure 3I through 3K). Left atrial contraction also remained similar (Figure 3L). There were no significant changes in left atrial volume during the trial (Figure S3D).

Similarly in study 2, treatment with ninerafaxstat was not associated with changes in LV end-diastolic volume, SV, mass, or LVEF (Figure 3M through 3Q). In study 2, although during exercise, ninerafaxstat treatment resulted in no change in LV end-diastolic volume, SV, or LV cardiac output; there was a significant increase in LV systolic augmentation (rest to exercise), with both LV cardiac output change and SV change (Figure S4A and S4B) being greater after treatment. This improvement in LVSV during exercise was correlated with improvement in PCr/ATP (Figure S4C), as was change in LVEF augmentation. Improvements were also seen in the right ventricle, with right ventricular (RV) augmentation exercise (rest to exercise) improved with treatment, with both RV cardiac output (Figure S4D) and right ventricular SV change with exercise (Figure S4E) being greater. Again, this improvement in SV during exercise was correlated with improvement in PCr/ATP (Figure S4F). No change in exercise diastology by echo was observed (data not shown). Greater improvement in PCr/ATP was associated with reduced lung water transudation during exercise (Figure S4G), which, itself, was associated with a greater change in 6-minute walk distance (Figure S4H). A greater augmentation of systolic function was associated with greater improvement in patient symptoms scores (Figure S4I through S4K).

#### Right Ventricle

In study 1, right ventricle size and systolic function were within normal limits at baseline and unchanged with treatment (Figure S3A through S3C). Similarly, in study 2, after treatment, resting RV end-diastolic volume and right ventricular end-systolic volume were smaller, with resting RV systolic function unchanged (Figure S3E through S3H).

Overall, treatment with ninerafaxstat was associated with improved PCr/ATP ratio, altered pyruvate metabolism, decreased MTGC, and changes in the circulating metabolome and proteome. Treatment with ninerafaxstat was also related to improved readouts of diastolic function.

### Health and Functional Status in HFpEF

Treatment with ninerafaxstat was associated with an improvement in patients’ global impression of severity score (Figure S5A), NYHA class (Figure S5B), and Kansas City cardiomyopathy questionnaire clinical summary score was numerically improved (Figure S5D). This improvement was accompanied by a 14-m increase in 6-minute walk distance (Figure S5C). In addition, greater change in lung water transudation with exercise after the trial was related to greatest improvement in Kansas City cardiomyopathy questionnaire clinical summary score (Figure S5E), quality of life score (Figure S5F), and change of total symptom score (Figure S5G).

Overall, in patients with cardiometabolic HFpEF, treatment with ninerafaxstat improved myocardial PCr/ATP and improved systolic augmentation during exercise. Myocardial triglyceride increased during the study.

### Safety and Tolerability

Ninerafaxstat was safe and well tolerated. Of 22 participants in part 1, 13 (≈59%) reported 15 cumulative treatment-emergent adverse events, all of which were described as mild or moderate in severity and self-limiting. Five AEs (one mild and 4 moderate) were judged to be related to the study drug, with one participant developing diarrhea and dizziness (both moderate in severity) after 2 days of dosing and electing to withdraw from the study. In part 2, of the 26 participants who received ninerafaxstat, 13 participants (48%) reported 39 treatment-emergent AEs, all of which were described as mild to moderate in severity and self-limiting. Sixteen AEs (12 mild and 4 moderate) were judged to be related to the study drug. Three participants withdrew from the study drug because of gastrointestinal side effects (nausea, bloating, or diarrhea). Two were excluded because of <80% compliance (prespecified in the study protocol) and one because of a rash after 2 months of treatment. There was one treatment-emergent serious AE attributable to hospital admission with recurrent back pain, which was judged not to be related to the study drug.

## DISCUSSION

The heart in diabetes and obesity is characterized by increased FAO and reduced PDH flux.^16^ This overreliance on FAO for ATP generation and the reduced “metabolic flexibility” results in an energy deficit, cardiac steatosis, LV hypertrophy, and LV diastolic dysfunction.^34^ This has been put forward as 1 of the mechanisms of why this population is susceptible to HFpEF.

Our study presented here was split into two parts. In part 1 we evaluated the impact of the novel metabolic modulator ninerafaxstat in participants with T2D and obesity without HFpEF. We observed improved myocardial energetics (PCr/ATP) and altered pyruvate metabolism after treatment. Furthermore, ninerafaxstat reduced myocardial steatosis and improved diastolic function. As this suggested potential for benefit in more advanced diabetic heart disease, part 2 evaluated the impact of ninerafaxstat on myocardial energetics, metabolism, and function in participants with T2D, obesity, and HFpEF. This again showed improved resting PCr/ATP but also improved biventricular systolic augmentation during exercise, with further subjective improvement in 6-minute walk distance and NYHA class symptoms. In contrast to findings in part 1, myocardial triglyceride was increased during the study.

### Effect of Ninerafaxstat on Myocardial Metabolism

The energetic status of the heart, measured by PCr/ATP, is lower in patients with T2D/obesity and HF,^20,35^ correlates with NYHA class, and independently predicts all-cause and cardiovascular mortality.^36^ In both parts of this study, ninerafaxstat treatment was associated with significant improvement in resting myocardial PCr/ATP. Upstream of ATP generation, PDH flux controls the balance of glucose and FAO, is blunted in HF, and has been proposed as a target for improving energy deficiency in diabetic heart disease.^37,38^ We observed that treatment with ninerafaxstat increased the [^13^C]bicarbonate/[1-^13^C]pyruvate ratio in 7 of 9 participants and reduced transamination of pyruvate to alanine without altering exchange through lactate dehydrogenase. These findings support the proposed mechanism of action of ninerafaxstat as a cardiac mitotrope to increase PDH flux, restore glycolysis/glucose oxidation coupling, and improve mitochondrial energetics. T2D, obesity, and HFpEF are linked to increased myocardial lipid deposition (steatosis), associated with LV remodeling and contractile and diastolic dysfunction.^34,39–41^ In part 1, among participants with T2D, ninerafaxstat nearly universally reduced MTGC. Although weight loss can reduce MTGC,^42–45^ only modest weight loss (median 0.8 kg) occurred, suggesting direct drug effects. In part 2, in HFpEF, MTGC increased slightly, consistent with partial FAO inhibition but without associated diastolic dysfunction, energetic compromise, or remodeling, suggesting minimal clinical relevance of this increase.

### Cardiac Functional and Symptomatic Improvements

In part 1, in participants with obesity and T2D, treatment with ninerafaxstat improved several markers of LV diastolic function. As this occurred without a change in LV mass and in the face of improved PCr/ATP and reduced MTGC, it is likely to represent a change in an active (metabolic), rather than passive (structural) mechanism. In part 2, in participants with HFpEF, we observed an improvement in biventricular systolic augmentation during exercise, which was associated with improved PCr/ATP. As both diastole and systole are energy-demanding states, this improvement in myocardial energetics is likely to be an important contributor to functional improvements seen in both studies. Although traditionally, HFpEF is regarded as primarily a problem with diastole, it has been shown almost unanimously that reduced contractile reserve during exercise is also a central feature of HFpEF.^46,47^ We have previously shown that this failure of contractile reserve is linked to reduced PCr/ATP.^20^ In obesity, weight loss interventions have improved both PCr/ATP and diastolic function,^15,45^ whereas in diabetes, SGLT2i have been shown to improve PCr/ATP and LVEF,^48^ similar to TMZ in dilated cardiomyopathy.^49^ On the contrary, perhexiline improves PCr/ATP early in dilated cardiomyopathy without a change in LVEF.^50^

Here, we have shown that the improved PCr/ATP with ninerafaxstat is associated with improved systolic reserve. Importantly, this improved systolic augmentation was also related to improvements in symptoms and quality of life.

### Comparison of Ninerafaxstat With Other Metabolic Treatments

Recent mechanistic studies of SGLT2i in HFpEF have demonstrated beneficial effects on LV filling pressures and stress RV function as well as favorable changes in body composition and visceral fat.^51,52^ SGLT2i have also been shown to improve PCr/ATP in diabetes,^48^ with change seen in HFpEF.^53^ Interestingly, many of these findings parallel observations in this study, including improvements in exercise-induced systolic augmentation, RV functional reserve, and symptomatic status with ninerafaxstat treatment. Unlike SGLT2i, which have shown modest reductions in NT-proBNP and improvements in glucose-insulin homeostasis,^54^ treatment with ninerafaxstat did not significantly alter the glycemic index, lipid profile, or NT-proBNP. Together with the evidence that SGLT2i reduce myocardial glucose uptake^55^ without altering fatty acid uptake or oxidation,^56^ this suggests a metabolically distinct mechanism of action, potentially more directly linked to myocardial energy metabolism.

GLP-1 (glucagon-like peptide 1) receptor agonism has also shown similar results, rapidly improving PCr/ATP, and hyperpolarized ^13^C-MRS measured PDH flux in a rodent model of obesity without a change in weight.^37^ GLP-1 agonism also improves PCr/ATP in patients with T2D.^57^ Although GLP-1 agonists have not been evaluated mechanistically in human HFpEF, improvements appear to be mediated mainly indirectly by associated weight loss, which, itself, improves PCr/ATP.^45^ In contrast, ninerafaxstat increased the myocardial PCr/ATP ratio in T2D and HFpEF without a change in circulating substrates or significant weight loss. As improvements in exercise hemodynamics correlated with enhanced myocardial energetics rather than systemic substrate shifts or volume changes, this suggests a potential additive role for ninerafaxstat in patients with energy-starved HFpEF phenotypes.

### Comparison of Ninerafaxstat With TMZ

Given the mechanism of action, the recent trial of TMZ in HFpEF is worth discussing.^58^ This showed that TMZ in all comers with HFpEF did not improve myocardial PCr/ATP or exercise hemodynamics in participants with HFpEF. This differs from this trial, in which ninerafaxstat improved PCr/ATP along with exercise systolic function in participants with obesity, T2D, and nonischemic HFpEF. Compared with the recent TMZ trial, this study included only participants with diabetes and excluded those with coronary artery disease, whereas 43% of the TMZ cohort had coronary disease. Participants in this study had higher BMI and NT-proBNP and greater use of β-blockers, renin-angiotensin-aldosterone system inhibitors, and anticoagulants. Additionally, the ^31^P-MRS methods differed substantially. In the TMZ study, a whole-heart voxel was used without saturation bands, whereas in this study, a smaller, septal-only 3D localized voxel was used, with additional saturation bands to minimize skeletal muscle and liver signal contamination. Therefore, the populations, drugs, and techniques of these 2 studies alone are sufficiently different to explain the difference in results.

The greater efficacy of ninerafaxstat compared with generic TMZ can be attributed to the additive pharmacological effects of its 3 active moieties (IMB-1028814, TMZ, and carboxy-8814), which collectively provide an ≈3-fold higher systemic exposure in terms of TMZ equivalents relative to the approved dose of TMZ. In addition, each 200-mg MR capsule comprises ≈29% niacin (58.8 mg). By reducing peripheral fatty acid levels and increasing GLUT4 expression,^59^ this increases myocardial glucose uptake (a level comparable to insulin-glucose clamp).^60^ When combined with partial FAO inhibition of the 3 active moieties, ninerafaxstat enhances the coupling of increased glucose uptake with oxidation via PDH. In addition to this, niacin is a systemically bioavailable, cell-permeant NAD^+^ precursor. The heart lacks the enzymes needed for de novo biosynthesis of NAD^+^, a critical cofactor for redox reactions, mitochondrial bioenergetics and function (including via protein lysine acetylation), cellular response to oxidative stress, and metabolic flexibility. Cardiac NAD^+^ levels are reduced in cardiac tissue from patients with HFpEF, with higher dietary niacin and niacin equivalent intake associated with lower risk of HFpEF and cardiac death in humans.^61^

Notably, this magnitude of improvement in PCr/ATP is greater than that described with SGLT2i in T2D.^48^ In part 1, PCr/ATP at the end of treatment recovered to within the physiologically normal range, consistent with enhanced cardiac energy reserves.^34^ In participants with HFpEF, PCr/ATP also improved by 12%. Although the magnitude of change is smaller, this is one of the very few studies to demonstrate enhanced cardiac energetics in HF and the only study to do so in HFpEF, with other studies showing improvement with medical therapies for heart failure wtih reduced ejection fraction, such as beta-blockers^62^ and perhexiline.^50^

### Study Limitations

We provide a detailed assessment of metabolic changes induced by treatment with ninerafaxstat, using not only multinuclear MRS (^31^P and ^1^H) but also hyperpolarized [1-^13^C]pyruvate MRS and plasma metabolomics and proteomics. Furthermore, the additional use of MR imaging, the gold-standard imaging modality to assess cardiac function and structure, increases the reliability of our findings. Nevertheless, limitations of our studies include a relatively small sample size, open-label design, no placebo group, and recruitment at a single center. Female and male participants were not entirely equal in our cohort, and all participants were ethnically White.

Part 1 involved stress ^31^P-MRS, for which dobutamine was used to reduce thoracic motion during imaging. In part 2, exercise was used instead. Although both stressors induce adrenergically driven increases in heart rate and SV—and similar heart rates were achieved in this study—the differences in stress modality should be considered when interpreting comparisons between the 2 parts.

As the metabolic fluxes of PDH, lactate dehydrogenase, and alanine aminotransferase were inferred from the summed peaks of bicarbonate, lactate, and alanine normalized to pyruvate (presented as a ratio), any change in pyruvate in the blood pool can influence the ratio, independent of metabolism. As ninerafaxstat treatment was not associated with a reduction in ventricular cavity size, and blood pool pyruvate levels were not significantly lower after treatment, the observed increase in bicarbonate/pyruvate cannot be accounted for by blood pool pyruvate changes. The decrease in alanine/pyruvate also cannot be explained by a reduction in the pyruvate pool. These both point strongly to metabolic changes in pyruvate fate being the primary explanation for the changes seen.

A limitation of this study is the low use of contemporary pharmacotherapy for HFpEF, particularly SGLT2i and GLP-1 receptor agonists, among participants. During the study period (January 2023–June 2024), National Institute for Health and Care Excellence approval for dapagliflozin (TA902), empagliflozin (TA929), and semaglutide (TA875) occurred, but real-world clinical uptake was still emerging. Consequently, treatment patterns may not fully reflect current guideline-directed therapy, particularly in participants with HFpEF, obesity, and T2D. This limitation reflects the timing of recruitment during the early market adoption phase of these therapies.

Female representation was low in part 2 of our study (29%), which is notable given the well-documented sex differences in myocardial metabolism. Women typically show higher myocardial glucose uptake and greater metabolic flexibility, which may influence both disease phenotype and response to therapies like ninerafaxstat. This underrepresentation is also noteworthy because women make up the majority of HFpEF cases, particularly in obesity-related subtypes, comprising ≥60% of patients in large trials such as TOPCAT and I-PRESERVE.

### Summary

In participants with T2D and obesity but without HFpEF, the heart was characterized by impaired PDH flux, reduced PCr/ATP, triglyceride deposition, and diastolic impairment. Treatment with ninerafaxstat was associated with improved PDH flux, improved PCr/ATP, reduced myocardial TG, and improved diastolic function. In participants with T2D, obesity, and symptomatic HFpEF, the heart was characterized by reduced PCr/ATP, diastolic impairment, and failure of systolic augmentation to exercise. Treatment with ninerafaxstat was associated with improved PCr/ATP, improved biventricular systolic augmentation to exercise, improved exercise capacity and HF symptoms, and reduced NYHA class. This 2-part early mechanistic phase 2a study shows that ninerafaxstat is safely tolerated across the spectrum of cardiometabolic heart disease and may have energetic and functional benefits in the treatment of cardiometabolic HFpEF.

## ARTICLE INFORMATION

### Acknowledgments

The authors gratefully acknowledge the participants in this study for their time and patience and express their gratitude toward the OCMR research nursing team for their support in conducting this study, especially Judith Delossantos.

### Sources of Funding

The trial was funded by Imbria Pharmaceuticals and coordinated by Medpace (a contract research organization). Dr Hundertmark is supported by a protected research time grant funded by the Faculty of Medicine, University of Bern, Switzerland and acknowledges project-specific support by Imbria Pharmaceuticals. Dr Rider is primarily funded by a British Heart Foundation senior clinical research fellowship (FS/SCRF/22/32014). Dr Valkovič is supported by the Sir Henry Dale fellowship, jointly funded by the Royal Society and the Wellcome Trust (221805/Z/20/Z), and also acknowledges support of the Slovak Grant Agencies Vedecká Grantová Agentúra (2/0004/23) and Slovak Research and Development Agency (21-0299). Dr Miller acknowledges support from the Novo Foundation (reference NNF21OC0068683) and a Novo Nordisk postdoctoral fellowship run in conjunction with the University of Oxford. The views expressed are those of the authors and not necessarily those of the National Health Service, the National Institute for Health and Care Research or the Department of Health and Social Care. Dr Lewis acknowledges funding from the British Heart Foundation Oxford Centre for Research Excellence (RE/18/3/34214), the Oxford National Institute for Health and Care Research Biomedical Research Centre, and the British Heart Foundation (FS/ICRF/24/26111). Dr Neubauer acknowledges support from the Oxford National Institute for Health and Care Research Biomedical Research Centre and the British Heart Foundation Centre of Research Excellence.

### Disclosures

Drs Chamberlin and Patel are employees of Imbria Pharmaceuticals who supported development of the trial protocol but did not participate in the experiments or writing of this manuscript. Dr Dehbi is a consultant to Imbria Pharmaceuticals and Weatherden Ltd. Dr Sarwar is an employee of the University of Oxford and consultant to Imbria Pharmaceuticals, Weatherden Ltd, and Ultromics Ltd. Dr Yavari is an employee of the University of Oxford and Weatherden Ltd and a consultant to Imbria Pharmaceuticals.

## Supplemental Material

Table S1–S6

Figures S1–S5

Supplemental Methods

Reference 63

## Supplementary Material

## References

[1] Ng ACT Delgado V Borlaug BA Bax JJ. Diabesity: the combined burden of obesity and diabetes on heart disease and the role of imaging. Nat Rev Cardiol. 2021;18:291–304. doi: 10.1038/s41569-020-00465-5 33188304 10.1038/s41569-020-00465-5

[2] Seferovic PM Petrie MC Filippatos GS Anker SD Rosano G Bauersachs J Paulus WJ Komajda M Cosentino F de Boer RA. Type 2 diabetes mellitus and heart failure: a position statement from the Heart Failure Association of the European Society of Cardiology. Eur J Heart Fail. 2018;20:853–872. doi: 10.1002/ejhf.1170 29520964 10.1002/ejhf.1170

[3] Rubler S Dlugash J Yuceoglu YZ Kumral T Branwood AW Grishman A. New type of cardiomyopathy associated with diabetic glomerulosclerosis. Am J Cardiol. 1972;30:595–602. doi: 10.1016/0002-9149(72)90595-4 4263660 10.1016/0002-9149(72)90595-4

[4] Stanton AM Vaduganathan M Chang LS Turchin A Januzzi JL Jr. Aroda VR. Asymptomatic diabetic cardiomyopathy: an underrecognized entity in type 2 diabetes. Curr Diab Rep. 2021;21:41. doi: 10.1007/s11892-021-01407-2 34580767 10.1007/s11892-021-01407-2

[5] Dunlay SM Givertz MM Aguilar D Allen LA Chan M Desai AS Deswal A Dickson VV Kosiborod MN Lekavich CL; American Heart Association Heart Failure and Transplantation Committee of the Council on Clinical Cardiology; Council on Cardiovascular and Stroke Nursing; and the Heart Failure Society of America. Type 2 diabetes mellitus and heart failure: a scientific statement from the American Heart Association and the Heart Failure Society of America: This statement does not represent an update of the 2017 ACC/AHA/HFSA heart failure guideline update. Circulation. 2019;140:e294–e324. doi: 10.1161/CIR.0000000000000691 31167558 10.1161/CIR.0000000000000691

[6] Jia G Hill MA Sowers JR. Diabetic cardiomyopathy: an update of mechanisms contributing to this clinical entity. Circ Res. 2018;122:624–638. doi: 10.1161/CIRCRESAHA.117.311586 29449364 10.1161/CIRCRESAHA.117.311586 PMC5819359

[7] Neubauer S. The failing heart--an engine out of fuel. N Engl J Med. 2007;356:1140–1151. doi: 10.1056/NEJMra063052 17360992 10.1056/NEJMra063052

[8] Fukushima A Lopaschuk GD. Cardiac fatty acid oxidation in heart failure associated with obesity and diabetes. Biochim Biophys Acta. 2016;1861:1525–1534. doi: 10.1016/j.bbalip.2016.03.020 26996746 10.1016/j.bbalip.2016.03.020

[9] Lopaschuk GD Karwi QG Tian R Wende AR Abel ED. Cardiac energy metabolism in heart failure. Circ Res. 2021;128:1487–1513. doi: 10.1161/CIRCRESAHA.121.318241 33983836 10.1161/CIRCRESAHA.121.318241 PMC8136750

[10] Chong CR Clarke K Levelt E. Metabolic remodeling in diabetic cardiomyopathy. Cardiovasc Res. 2017;113:422–430. doi: 10.1093/cvr/cvx018 28177068 10.1093/cvr/cvx018 PMC5412022

[11] Schiattarella GG Rodolico D Hill JA. Metabolic inflammation in heart failure with preserved ejection fraction. Cardiovasc Res. 2021;117:423–434. doi: 10.1093/cvr/cvaa217 32666082 10.1093/cvr/cvaa217 PMC8599724

[12] Tan Y Zhang Z Zheng C Wintergerst KA Keller BB Cai L. Mechanisms of diabetic cardiomyopathy and potential therapeutic strategies: preclinical and clinical evidence. Nat Rev Cardiol. 2020;17:585–607. doi: 10.1038/s41569-020-0339-2 32080423 10.1038/s41569-020-0339-2 PMC7849055

[13] Huang JM Xian H Bacaner M. Long-chain fatty acids activate calcium channels in ventricular myocytes. Proc Natl Acad Sci U S A. 1992;89:6452–6456. doi: 10.1073/pnas.89.14.6452 1321440 10.1073/pnas.89.14.6452 PMC49519

[14] Scheuermann-Freestone M Madsen PL Manners D Blamire AM Buckingham RE Styles P Radda GK Neubauer S Clarke K. Abnormal cardiac and skeletal muscle energy metabolism in patients with type 2 diabetes. Circulation. 2003;107:3040–3046. doi: 10.1161/01.CIR.0000072789.89096.10 12810608 10.1161/01.CIR.0000072789.89096.10

[15] Rayner JJ Peterzan MA Watson WD Clarke WT Neubauer S Rodgers CT Rider OJ. Myocardial energetics in obesity: enhanced ATP delivery through creatine kinase with blunted stress response. Circulation. 2020;141:1152–1163. doi: 10.1161/CIRCULATIONAHA.119.042770 32138541 10.1161/CIRCULATIONAHA.119.042770 PMC7144750

[16] Rider OJ Apps A Miller J Lau JYC Lewis AJM Peterzan MA Dodd MS Lau AZ Trumper C Gallagher FA. Noninvasive in vivo assessment of cardiac metabolism in the healthy and diabetic human heart using hyperpolarized ^13^C MRI. Circ Res. 2020;126:725–736. doi: 10.1161/CIRCRESAHA.119.316260 32078413 10.1161/CIRCRESAHA.119.316260 PMC7077975

[17] Levelt E Rodgers CT Clarke WT Mahmod M Ariga R Francis JM Liu A Wijesurendra RS Dass S Sabharwal N. Cardiac energetics, oxygenation, and perfusion during increased workload in patients with type 2 diabetes mellitus. Eur Heart J. 2016;37:3461–3469. doi: 10.1093/eurheartj/ehv442 26392437 10.1093/eurheartj/ehv442 PMC5201143

[18] Levelt E Pavlides M Banerjee R Mahmod M Kelly C Sellwood J Ariga R Thomas S Francis J Rodgers C. Ectopic and visceral fat deposition in lean and obese patients with type 2 diabetes. J Am Coll Cardiol. 2016;68:53–63. doi: 10.1016/j.jacc.2016.03.597 27364051 10.1016/j.jacc.2016.03.597 PMC4925621

[19] van de Weijer T Schrauwen-Hinderling VB Schrauwen P. Lipotoxicity in type 2 diabetic cardiomyopathy. Cardiovasc Res. 2011;92:10–18. doi: 10.1093/cvr/cvr212 21803867 10.1093/cvr/cvr212

[20] Burrage MK Hundertmark M Valkovic L Watson WD Rayner J Sabharwal N Ferreira VM Neubauer S Miller JJ Rider OJ. Energetic basis for exercise-induced pulmonary congestion in heart failure with preserved ejection fraction. Circulation. 2021;144:1664–1678. doi: 10.1161/CIRCULATIONAHA.121.054858 34743560 10.1161/CIRCULATIONAHA.121.054858 PMC8601674

[21] Mahmod M Pal N Rayner J Holloway C Raman B Dass S Levelt E Ariga R Ferreira V Banerjee R. The interplay between metabolic alterations, diastolic strain rate and exercise capacity in mild heart failure with preserved ejection fraction: a cardiovascular magnetic resonance study. J Cardiovasc Magn Reson. 2018;20:88. doi: 10.1186/s12968-018-0511-6 30580760 10.1186/s12968-018-0511-6 PMC6304764

[22] Chamberlin P Harding M Patel J Levin A. IMB-1018792, a novel first-in class partial fatty acid oxidation inhibitor improves cardiac remodeling and function post-myocardial infarction. J Am Coll Cardiol. 2021;77:539–539. doi: 10.1016/s0735-1097(21)01898-2

[23] Maron MS Mahmod M Abd Samat AH Choudhury L Massera D Phelan DMJ Cresci S Martinez MW Masri A Abraham TP. Safety and efficacy of metabolic modulation with ninerafaxstat in patients with nonobstructive hypertrophic cardiomyopathy. J Am Coll Cardiol. 2024;83:2037–2048. doi: 10.1016/j.jacc.2024.03.387 38599256 10.1016/j.jacc.2024.03.387

[24] Pieske B Tschöpe C de Boer RA Fraser AG Anker SD Donal E Edelmann F Fu M Guazzi M Lam CSP. How to diagnose heart failure with preserved ejection fraction: the HFA–PEFF diagnostic algorithm: a consensus recommendation from the Heart Failure Association (HFA) of the European Society of Cardiology (ESC). Eur Heart J. 2019;40:3297–3317. doi: 10.1093/eurheartj/ehz641 31504452 10.1093/eurheartj/ehz641

[25] Sueta D Yamamoto E Nishihara T Tokitsu T Fujisue K Oike F Takae M Usuku H Takashio S Arima Y. H2FPEF score as a prognostic value in HFpEF patients. Am J Hypertens. 2019;32:1082–1090. doi: 10.1093/ajh/hpz108 31271191 10.1093/ajh/hpz108

[26] Schulz-Menger J Bluemke DA Bremerich J Flamm SD Fogel MA Friedrich MG Kim RJ von Knobelsdorff-Brenkenhoff F Kramer CM Pennell DJ. Standardized image interpretation and post-processing in cardiovascular magnetic resonance - 2020 update: Society for Cardiovascular Magnetic Resonance (SCMR): Board of Trustees Task Force on Standardized Post-Processing. J Cardiovasc Magn Reson. 2020;22:19. doi: 10.1186/s12968-020-00610-6 32160925 10.1186/s12968-020-00610-6 PMC7066763

[27] Tyler DJ Emmanuel Y Cochlin LE Hudsmith LE Holloway CJ Neubauer S Clarke K Robson MD. Reproducibility of 31P cardiac magnetic resonance spectroscopy at 3 T. NMR Biomed. 2009;22:405–413. doi: 10.1002/nbm.1350 19023865 10.1002/nbm.1350

[28] Purvis LAB Clarke WT Biasiolli L Valkovič L Robson MD Rodgers CT. OXSA: An open-source magnetic resonance spectroscopy analysis toolbox in MATLAB. PLoS One. 2017;12:e0185356. doi: 10.1371/journal.pone.0185356 28938003 10.1371/journal.pone.0185356 PMC5609763

[29] Rial B Robson MD Neubauer S Schneider JE. Rapid quantification of myocardial lipid content in humans using single breath-hold ^1^H MRS at 3 Tesla. Magn Reson Med. 2011;66:619–624. doi: 10.1002/mrm.23011 21721038 10.1002/mrm.23011 PMC3427889

[30] Banerjee R Pavlides M Tunnicliffe EM Piechnik SK Sarania N Philips R Collier JD Booth JC Schneider JE Wang LM. Multiparametric magnetic resonance for the non-invasive diagnosis of liver disease. J Hepatol. 2014;60:69–77. doi: 10.1016/j.jhep.2013.09.002 24036007 10.1016/j.jhep.2013.09.002 PMC3865797

[31] Tahir UA Katz DH Zhao T Ngo D Cruz DE Robbins JM Chen ZZ Peterson B Benson MD Shi X. Metabolomic profiles and heart failure risk in Black adults: insights from the Jackson Heart Study. Circ Heart Fail. 2021;14:e007275. doi: 10.1161/CIRCHEARTFAILURE.120.007275 33464957 10.1161/CIRCHEARTFAILURE.120.007275 PMC9158510

[32] Assarsson E Lundberg M Holmquist G Bjorkesten J Thorsen SB Ekman D Eriksson A Rennel Dickens E Ohlsson S Edfeldt G. Homogenous 96-plex PEA immunoassay exhibiting high sensitivity, specificity, and excellent scalability. PLoS One. 2014;9:e95192. doi: 10.1371/journal.pone.0095192 24755770 10.1371/journal.pone.0095192 PMC3995906

[33] Hundertmark MJ Agbaje OF Coleman R George JT Grempler R Holman RR Lamlum H Lee J Milton JE Niessen HG. Design and rationale of the EMPA-VISION trial: investigating the metabolic effects of empagliflozin in patients with heart failure. ESC Heart Fail. 2021;8:2580–2590. doi: 10.1002/ehf2.13406 33960149 10.1002/ehf2.13406 PMC8318430

[34] Levelt E Mahmod M Piechnik SK Ariga R Francis JM Rodgers CT Clarke WT Sabharwal N Schneider JE Karamitsos TD. Relationship between left ventricular structural and metabolic remodeling in type 2 diabetes. Diabetes. 2016;65:44–52. doi: 10.2337/db15-0627 26438611 10.2337/db15-0627 PMC4890658

[35] Rider OJ Francis JM Ali MK Holloway C Pegg T Robson MD Tyler D Byrne J Clarke K Neubauer S. Effects of catecholamine stress on diastolic function and myocardial energetics in obesity. Circulation. 2012;125:1511–1519. doi: 10.1161/CIRCULATIONAHA.111.069518 22368152 10.1161/CIRCULATIONAHA.111.069518

[36] Neubauer S Horn M Cramer M Harre K Newell JB Peters W Pabst T Ertl G Hahn D Ingwall JS. Myocardial phosphocreatine-to-ATP ratio is a predictor of mortality in patients with dilated cardiomyopathy. Circulation. 1997;96:2190–2196. doi: 10.1161/01.cir.96.7.2190 9337189 10.1161/01.cir.96.7.2190

[37] Lewis AJM Dodd MS Sourdon J Lygate CA Clarke K Neubauer S Tyler DJ Rider OJ. Hyperpolarized (13)C and (31)P MRS detects differences in cardiac energetics, metabolism, and function in obesity, and responses following treatment. NMR Biomed. 2024;37:e5206. doi: 10.1002/nbm.5206 38994722 10.1002/nbm.5206 PMC11571269

[38] Lewis AJ Neubauer S Tyler DJ Rider OJ. Pyruvate dehydrogenase as a therapeutic target for obesity cardiomyopathy. Expert Opin Ther Targets. 2016;20:755–766. doi: 10.1517/14728222.2016.1126248 26617082 10.1517/14728222.2016.1126248

[39] Schulze PC Drosatos K Goldberg IJ. Lipid use and misuse by the heart. Circ Res. 2016;118:1736–1751. doi: 10.1161/CIRCRESAHA.116.306842 27230639 10.1161/CIRCRESAHA.116.306842 PMC5340419

[40] McGavock JM Lingvay I Zib I Tillery T Salas N Unger R Levine BD Raskin P Victor RG Szczepaniak LS. Cardiac steatosis in diabetes mellitus: a 1H-magnetic resonance spectroscopy study. Circulation. 2007;116:1170–1175. doi: 10.1161/CIRCULATIONAHA.106.645614 17698735 10.1161/CIRCULATIONAHA.106.645614

[41] Rijzewijk LJ van der Meer RW Smit JW Diamant M Bax JJ Hammer S Romijn JA de Roos A Lamb HJ. Myocardial steatosis is an independent predictor of diastolic dysfunction in type 2 diabetes mellitus. J Am Coll Cardiol. 2008;52:1793–1799. doi: 10.1016/j.jacc.2008.07.062 19022158 10.1016/j.jacc.2008.07.062

[42] Hammer S Snel M Lamb HJ Jazet IM van der Meer RW Pijl H Meinders EA Romijn JA de Roos A Smit JW. Prolonged caloric restriction in obese patients with type 2 diabetes mellitus decreases myocardial triglyceride content and improves myocardial function. J Am Coll Cardiol. 2008;52:1006–1012. doi: 10.1016/j.jacc.2008.04.068 18786482 10.1016/j.jacc.2008.04.068

[43] Rayner JJ Abdesselam I Peterzan MA Akoumianakis I Akawi N Antoniades C Tomlinson JW Neubauer S Rider OJ. Very low calorie diets are associated with transient ventricular impairment before reversal of diastolic dysfunction in obesity. Int J Obes (Lond). 2019;43:2536–2544. doi: 10.1038/s41366-018-0263-2 30464235 10.1038/s41366-018-0263-2 PMC6892735

[44] Rider OJ Francis JM Ali MK Petersen SE Robinson M Robson MD Byrne JP Clarke K Neubauer S. Beneficial cardiovascular effects of bariatric surgical and dietary weight loss in obesity. J Am Coll Cardiol. 2009;54:718–726. doi: 10.1016/j.jacc.2009.02.086 19679250 10.1016/j.jacc.2009.02.086

[45] Rider OJ Francis JM Tyler D Byrne J Clarke K Neubauer S. Effects of weight loss on myocardial energetics and diastolic function in obesity. Int J Cardiovasc Imaging. 2013;29:1043–1050. doi: 10.1007/s10554-012-0174-6 23269470 10.1007/s10554-012-0174-6

[46] Borlaug BA Olson TP Lam CS Flood KS Lerman A Johnson BD Redfield MM. Global cardiovascular reserve dysfunction in heart failure with preserved ejection fraction. J Am Coll Cardiol. 2010;56:845–854. doi: 10.1016/j.jacc.2010.03.077 20813282 10.1016/j.jacc.2010.03.077 PMC2950645

[47] Di Lisi D Ciampi Q Madaudo C Manno G Macaione F Novo S Novo G. Contractile reserve in heart failure with preserved ejection fraction. J Cardiovasc Dev Dis. 2022;9:248. doi: 10.3390/jcdd9080248 36005412 10.3390/jcdd9080248 PMC9409661

[48] Thirunavukarasu S Jex N Chowdhary A Hassan IU Straw S Craven TP Gorecka M Broadbent D Swoboda P Witte KK. Empagliflozin treatment is associated with improvements in cardiac energetics and function and reductions in myocardial cellular volume in patients with type 2 diabetes. Diabetes. 2021;70:2810–2822. doi: 10.2337/db21-0270 34610982 10.2337/db21-0270 PMC8660983

[49] Fragasso G Perseghin G De Cobelli F Esposito A Palloshi A Lattuada G Scifo P Calori G Del Maschio A Margonato A. Effects of metabolic modulation by trimetazidine on left ventricular function and phosphocreatine/adenosine triphosphate ratio in patients with heart failure. Eur Heart J. 2006;27:942–948. doi: 10.1093/eurheartj/ehi816 16510466 10.1093/eurheartj/ehi816

[50] Beadle RM Williams LK Kuehl M Bowater S Abozguia K Leyva F Yousef Z Wagenmakers AJ Thies F Horowitz J. Improvement in cardiac energetics by perhexiline in heart failure due to dilated cardiomyopathy. JACC Heart Fail. 2015;3:202–211. doi: 10.1016/j.jchf.2014.09.009 25650370 10.1016/j.jchf.2014.09.009

[51] Borlaug BA Reddy YNV Braun A Sorimachi H Omar M Popovic D Alogna A Jensen MD Carter R. Cardiac and metabolic effects of dapagliflozin in heart failure with preserved ejection fraction: the CAMEO-DAPA trial. Circulation. 2023;148:834–844. doi: 10.1161/CIRCULATIONAHA.123.065134 37534453 10.1161/CIRCULATIONAHA.123.065134 PMC10529848

[52] Naser JA Tada A Harada T Reddy YNV Carter RE Testani JM Jensen MD Borlaug BA. Effects of dapagliflozin on body composition and its relation to hemodynamics in heart failure with preserved ejection fraction. Circulation. 2024;149:2026–2028. doi: 10.1161/CIRCULATIONAHA.124.069479 38885297 10.1161/CIRCULATIONAHA.124.069479 PMC11185269

[53] Hundertmark MJ Adler A Antoniades C Coleman R Griffin JL Holman RR Lamlum H Lee J Massey D Miller J. Assessment of cardiac energy metabolism, function, and physiology in patients with heart failure taking empagliflozin: the randomized, controlled EMPA-VISION trial. Circulation. 2023;30:1654–1669. doi: 10.1161/CIRCULATIONAHA.122.062021 10.1161/CIRCULATIONAHA.122.062021 PMC10212585 37070436

[54] Li R Dai G Guan H Gao W Ren L Wang X Qu H. Scientific evidence of sodium-glucose cotransporter-2 inhibitors for heart failure with preserved ejection fraction: an umbrella review of systematic reviews and meta-analyses. Front Cardiovasc Med. 2023;10:1143658. doi: 10.3389/fcvm.2023.1143658 37252111 10.3389/fcvm.2023.1143658 PMC10213331

[55] Succurro E Vizza P Papa A Miceli S Cicone F Fiorentino TV Sciacqua A Andreozzi F Veltri P Cascini GL. Effects of 26 weeks of treatment with empagliflozin versus glimepiride on the myocardial glucose metabolic rate in patients with type 2 diabetes: the randomized, open-label, crossover, active-comparator FIORE trial. Diabetes Obes Metab. 2022;24:2319–2330. doi: 10.1111/dom.14816 35837991 10.1111/dom.14816 PMC9804559

[56] Lauritsen KM Nielsen BRR Tolbod LP Johannsen M Hansen J Hansen TK Wiggers H Moller N Gormsen LC Sondergaard E. SGLT2 inhibition does not affect myocardial fatty acid oxidation or uptake, but reduces myocardial glucose uptake and blood flow in individuals with type 2 diabetes: a randomized double-blind, placebo-controlled crossover trial. Diabetes. 2021;70:800–808. doi: 10.2337/db20-0921 33334875 10.2337/db20-0921

[57] Chowdhary A Thirunavukarasu S Joseph T Jex N Kotha S Giannoudi M Procter H Cash L Akkaya S Broadbent D. Liraglutide improves myocardial perfusion and energetics and exercise tolerance in patients with type 2 diabetes. J Am Coll Cardiol. 2024;84:540–557. doi: 10.1016/j.jacc.2024.04.064 39084829 10.1016/j.jacc.2024.04.064 PMC11296502

[58] van de Bovenkamp AA Geurkink KTJ Oosterveer FTP de Man FS Kok WEM Bronzwaer PNA Allaart CP Nederveen AJ van Rossum AC Bakermans AJ. Trimetazidine in heart failure with preserved ejection fraction: a randomized controlled cross-over trial. ESC Heart Fail. 2023;10:2998–3010. doi: 10.1002/ehf2.14418 37530098 10.1002/ehf2.14418 PMC10567667

[59] Penumathsa SV Thirunavukkarasu M Samuel SM Zhan L Maulik G Bagchi M Bagchi D Maulik N. Niacin bound chromium treatment induces myocardial Glut-4 translocation and caveolar interaction via Akt, AMPK and eNOS phosphorylation in streptozotocin induced diabetic rats after ischemia-reperfusion injury. Biochim Biophys Acta. 2009;1792:39–48. doi: 10.1016/j.bbadis.2008.10.018 19027847 10.1016/j.bbadis.2008.10.018

[60] Stone CK Holden JE Stanley W Perlman SB. Effect of nicotinic acid on exogenous myocardial glucose utilization. J Nucl Med. 1995;36:996–1002. 7769458

[61] Abdellatif M Trummer-Herbst V Koser F Durand S Adao R Vasques-Novoa F Freundt JK Voglhuber J Pricolo MR Kasa M. Nicotinamide for the treatment of heart failure with preserved ejection fraction. Sci Transl Med. 2021;13:1–15. doi: 10.1126/scitranslmed.abd7064 10.1126/scitranslmed.abd7064 PMC7611499 33568522

[62] Spoladore R Fragasso G Perseghin G De Cobelli F Esposito A Maranta F Calori G Locatelli M Lattuada G Scifo P. Beneficial effects of beta-blockers on left ventricular function and cellular energy reserve in patients with heart failure. Fundam Clin Pharmacol. 2013;27:455–464. doi: 10.1111/j.1472-8206.2012.01029.x 22320703 10.1111/j.1472-8206.2012.01029.x

[63] Tyler DJ Emmanuel Y Cochlin LE Hudsmith LE Holloway CJ Neubauer S Clarke K Robson MD. Reproducibility of 31P cardiac magnetic resonance spectroscopy at 3 T. NMR Biomed. 2009;22:405–413. doi: 10.1002/nbm.1350 19023865 10.1002/nbm.1350

