# Supplementary material for: IMPROVE-DiCE, a 2-Part, Open-Label, Phase 2a Trial Evaluating the Safety and Effectiveness of Ninerafaxstat in Patients With Cardiometabolic Syndromes
Source: Circulation. 2025 Dec 18;153(8):550–63. doi: 10.1161/CIRCULATIONAHA.125.074041 (PMC12928817; doi:10.1161/CIRCULATIONAHA.125.074041)
Supplement: Supplementary file 1 [file cir-153-550-s001.pdf]

SUPPLEMENTAL MATERIAL

Supplementary Table 1 – Extended Trial Inclusion and Exclusion Criteria

Inclusion Criteria

Part 1 and Part 2 Patients who met all of the following criteria were eligible to participate in the study:

1. Provision of written informed consent before any screening procedures;
2. Male or female aged ≥18 and ≤75 years (Part 1) or ≤80 years (Part 2) at screening;
3. Must agree to adequate contraception requirements as follows: a. WOCBP must have a negative serum pregnancy test at screening and a negative pregnancy test (serum or urine) on the day of baseline pre-dose; b. WOCBP must agree to use dual methods of contraception, including 1 highly effective and 1 effective method of contraception, from the day of first dosing until 3 months after the last administration of test product; and c. Male patients must use an effective barrier method of contraception if sexually active with a WOCBP, from the day of first dosing until 3 months after the last administration of test product
4. Must agree not to donate sperm or ova from the day of first dosing until 3 months after last dosing
5. Women not of childbearing potential must be either surgically sterile (hysterectomy, bilateral tubal ligation, salpingectomy, and/or bilateral oophorectomy at least 26 weeks before screening) or postmenopausal, defined as spontaneous amenorrhea for at least 2 years with follicle-stimulating hormone (FSH) in the postmenopausal range at screening
6. Must be able and willing to comply with all study procedures and requirements
7. Diagnosis of T2DM
8. Elevated HbA1c defined as ≥6.5% (≥48 mmol/mol)
9. Elevated BMI defined as ≥30 kg/m^2
10. Preserved LVEF (defined as ≥50%)
11. If on oral hypoglycaemic (anti-diabetic) therapy, no change in therapy over the past 3 months

Additional Inclusion Criteria for Part 2

12. Be ambulant and have clinically stable symptomatic (NYHA functional class II to III) HF
13. Diagnosis of HFpEF by the Heart Failure Association HFA-PEFF diagnostic algorithm or by obtaining a score of ≥6 in the H_2FPEF scoring system.

Exclusion Criteria

Part 1 and Part 2 Patients who meet any of the following criteria were excluded from participation in the study:

1. BMI >40 kg/m^2
2. Uncontrolled hypertension (defined as resting blood pressure >180/90 mmHg) at screening;
3. Standard contraindication(s) to MR scanning

4. More than mild to moderate (Part 1) or more than moderate (Part 2) valvular heart disease per Investigator's judgement; Note: For Part 2, patients with severe tricuspid regurgitation due to HFpEF may be eligible on a case-by case basis upon discussion with sponsor.
5. History of sustained ventricular tachycardia or cardiac arrest;
6. Active exertional angina or intermittent claudication;
7. Known significant obstructive coronary artery disease per Investigator's judgement;
8. Absolute or significant contraindication to dobutamine infusion, including: pheochromocytoma, LV outflow tract obstruction, untreated hyperthyroidism, severe hypotension, aortic dissection, or large aneurysm (Part 1 only);
9. History of stroke, transient ischaemic attack, acute coronary syndrome, myocardial infarction, peripheral vascular disease, diagnosis of NYHA functional class III or IV HF (excluded for Part 1 only), hospitalisation for HF, or any arterial revascularisation procedure (including coronary artery bypass grafting) within 6 months before screening (Part 1) or within 3 months before screening (Part 2);
10. Presence of indwelling cardiac device (pacemaker, cardiac resynchronisation therapy, and/or implantable cardioverter defibrillator);
11. Arrhythmia with an uncontrolled ventricular response >110 bpm (Stage 2 only);
12. Inability to exercise (Part 2 only);
13. Known significant primary lung disease or primary pulmonary hypertension, or uncontrolled obstructive sleep apnoea (Part 2 only);
14. Significant hepatic impairment defined as total bilirubin and/or ALT and/or aspartate aminotransferase (AST) >2 × upper limit of normal (ULN);
15. Moderate or severe renal impairment defined as estimated glomerular filtration rate (eGFR)
16. History of Parkinson disease, Parkinsonian symptoms, restless leg syndrome, or other related movement disorders;
17. Known allergy, intolerance, or absolute contraindication to TMZ or nicotinic acid;
18. Concomitant use within the last 1 month of TMZ, nicotinic acid (at prescription/therapeutic dose), perhexiline, meldonium, or ranolazine;
19. Any use of insulin and/or SGLT2 inhibitors (Part 1 only); Note: SGLT2 inhibitor use is permitted in Part 2 provided that the patient is on a stable dose for ≥3 months at the time of randomisation.
20. History of alcohol abuse or drug addiction within the previous 5 years;
21. Pregnant, or planning pregnancy or lactation;
22. Participation in another clinical study involving a test product or medical device within 28 days (or 5 half-lives of the test product, whichever is longer) prior to first dosing;
23. Any medical or surgical condition that may interfere with the patient's participation in the clinical study, significantly interfere with the interpretation of the results, or put the patient at significant risk, according to the Investigator's judgement, from study participation;
24. Known hypersensitivity to IMB-1018972, mannitol, hypromellose, magnesium stearate, or pre-gelatinised corn starch (other ingredients of placebo and active test product)

BMI=body mass index; FSH=follicle stimulating hormone; HbA1c=glycated haemoglobin A1c; IMB-1018972=ninerafaxstat; LVEF=left ventricular ejection fraction; SGLT2=sodium

glucose co-transporter 2; NYHA=New York Heart Association; T2DM=type 2 diabetes mellitus; WOCBP=women of childbearing potential

Supplementary Table 2 – Study medications for Study Part 1

DICE Study 1 Medication	Patients taking (of 21 completers)	Dose Changed
Indapamide	4	No
Bendroflumethiazide	1	No
Semaglutide	1	No
ACEi	7	No
ARB	6	No
Ca^{2+} blocker	9	No
Diltiazem	1	No
Doxazosin	2	No
Aspirin	2	No
Exenatide	1	No
Statin	15	No
Ezetimibe	1	No
Metformin	21	No
Gliclazide	5	No
Pioglitazone	1	No
Sitagliptin	1	No

Supplementary Table 3 – Study medications for Study Part 2

DICE Study 2 Medication:	Patients taking (of 21 completers)	Dose Changed
Furosemide	13	Yes*
Bendroflumethiazide	1	No
SGLT-2i	3	No
Semaglutide	1	Yes+
ACEi	16	No
ARB	1	No
MRA	6	No
Beta-Blocker	16	No
Ca^{2+} Blocker	8	No
Diltiazem	2	No
Flecainide	1	No
Digoxin	1	No
Doxazosin	2	No
Apixaban	14	No
Warfarin	1	No
Aspirin	4	No
Cloidogrel	1	No
Statin	19	No
Ezetimibe	1	No
Insulin	4	YES*
Metformin	9	No
Gliclazide	1	No

* average daily dose 35mg, one participant had a reduction from 40 to 20 furosemide, and a reduction in insulin

+ one participant was on Semaglutide and had a dose reduction from 1.5 to 0.5 due to GI side effects

Supplement Table 4 – Extended Anthropometrics and Blood Biomarkers for Study Part 1

IQR is defined as 25th percentile, 75th percentile

	Pre Treatment	Post Treatment	p
**Anthropometrics, mean (SD)**			
Weight (kg)	97 (12)	95 (12)	0.04
Body mass index (kg/m^2)	33.6 (3.6)	33.3 (3.8)	0.06
Systolic blood pressure (mmHg)	142 (15)	140 (13)	0.42
Diastolic blood pressure (mmHg)	73 (8)	75 (8)	0.58
Resting heart rate (bpm)	75 (11)	70 (10)	0.009
Heart rate during stress (bpm)	112 (10)	110 (6)	0.31
**Blood Biomarkers Mean (SD) or Median (IQR)**			
Total cholesterol (mmol/l)	4.3 (1.0)	4.2 (0.9)	0.08
LDL cholesterol (mmol/l)	2.4 (1.0)	2.2 (0.8)	0.02
HDL cholesterol (mmol/l)	1.2 (0.2)	1.2 (0.2)	0.84
Triglycerides (mmol/l)	1.6 (0.6)	1.7 (0.9)	0.46
Fasting glucose (mmol/l)	7.9 (1.8)	8.0 (2.4)	0.94
Fasting insulin (pmol/l)	86 (40)	84 (37)	0.51
HOMA-IR	4.6 (3.2)	5.1 (3.3)	0.56
Free Fatty Acids (mmol/l)	0.9 (0.2)	0.9 (0.3)	0.77
Albumin (g/l)	39 (2)	39 (2)	0.73
ALT (mmol/l)	28 (13)	28 (14)	0.84
AST (mmol/l)	23 (12)	22 (9)	0.33
GGT (mmol/l)	42 (34)	38 (24)	0.38
Bilirubin (mmol/l)	11 (4)	12 (5)	0.32
Amylase (mmol/l)	50 (14)	52 (14)	0.37
Urea (mmol/l)	2.3 (0.7)	2.6 (0.8)	0.01
Creatinine (mmol/l)	68 (15)	70 (16)	0.14
Potassium (mmol/l)	4.0 (0.2)	3.9 (0.2)	0.09
Sodium (mmol/l)	139 (2)	140 (2)	0.29
eGFR (mL/min/1.73m^2)	84 (8)	82 (10)	0.06
NT-pro BNP (ng/ml)	82 (59,147)	60 (40,109)	0.72
hs-cTnl (ng/l)	2 (2,5)	2 (2,4)	0.26
Creatine Kinase (mmol/l)	98 (105)	119 (110)	0.03
Chloride (mmol/l)	104 (3)	104 (3)	0.09
Calcium (mmol/l)	2.3 (0.1)	2.4 (0.1)	0.69
Phosphate (mmol/l)	1.0 (0.1)	1.0 (0.1)	0.69

Supplement Table 5 – Extended Anthropometrics and Blood Biomarkers for Study Part 2

	Pre Treatment	Post Treatment	p
**Anthropometrics, mean (SD)**			
Weight (kg)	104 (14)	103 (14)	0.08
Body mass index (kg/m^2)	35.2 (4.0)	34.8 (4.2)	0.04
Visceral Fat Mass (cm^2)	272 (94)	277 (98)	0.58
Systolic blood pressure (mmHg)	137 (18)	138 (23)	0.72
Diastolic blood pressure (mmHg)	77 (9)	75 (10)	0.2
Resting heart rate (bpm)	70 (13)	69 (15)	0.79
Heart rate during stress (bpm)	105 (28)	110 (23)	0.34
**Blood Biomarkers Mean (SD) or Median (IQR)**			
Total cholesterol (mmol/l)	4.0 (1.0)	3.9 (0.9)	0.49
LDL cholesterol (mmol/l)	2.2 (0.3)	2.1 (0.7)	0.24
HDL cholesterol (mmol/l)	1.2 (0.9)	1.2 (0.8)	0.87
Triglycerides (mmol/l)	1.6 (1.0)	1.5 (1.2)	0.53
Fasting glucose (mmol/l)	6.7 (1.3)	7.0 (1.9)	0.25
Fasting insulin (pmol/l)	116 (101)	111 (68)	0.77
HOMA-IR	5.0 (4.7)	5.1 (3.9)	0.86
Free Fatty Acids (mmol/l)	0.6 (0.3)	0.6 (0.3)	0.72
BOHB (mmol/l)	0.2 (0.2)	0.1 (0.1)	0.45
Urate (umol/l)	409 (87)	412 (75)	0.69
Albumin (g/l)	41 (4)	41 (4)	0.48
ALT (mmol/l)	27 (15)	29 (18)	0.37
AST (mmol/l)	27 (9)	28 (10)	0.50
GGT (mmol/l)	93 (178)	94 (182)	0.95
Bilirubin (mmol/l)	13 (5)	13 (6)	0.47
Amylase (mmol/l)	59 (37)	66 (42)	0.09
Urea (mmol/l)	3.6 (0.9)	3.7 (1.2)	0.62
Creatinine (mmol/l)	95 (49)	92 (48)	0.80
Potassium (mmol/l)	4.5 (0.4)	4.4 (0.4)	0.22
Sodium (mmol/l)	140 (2)	140 (3)	0.35
eGFR (mL/min/1.73m^2)	66 (16)	65 (18)	0.28
NT-pro BNP (ng/ml)	538 (246,1222)	679 (211,1147)	0.56
hs-cTn (ng/l)	5 (3,9.5)	4.5 (2.25,8.5)	0.16
Creatine Kinase (mmol/l)	94 (48)	92 (48)	0.8
Chloride (mmol/l)	104 (3)	104 (4)	0.91
Calcium (mmol/l)	2.3 (0.1)	2.3 (0.1)	0.72
Phosphate (mmol/l)	1.1 (0.2)	1.1 (0.2)	0.28
CRP (mg/l)	5 (5)	7 (9)	0.15

Study Part 1

	Pre	post	p
E/A (SR only)	0.84 (0.74, 0.95)	0.77 (0.67, 0.93)	0.84
E/e lateral	7.3 (5.6, 8.5)	8.0 (5.8, 13.75)	0.02
Average E/E’	8.9 (7.2, 10.0)	9.1 (7.4,12.3)	0.07
Peak Diastolic Strain Rate Circ (1/s)	0.86 (0.82, 1.06)	0.99 (0.90, 1.09)	**0.047**
Peak LV Filling Rate (ml/s)	336 (290, 452)	373 (312, 520)	0.06
Normalised Peak LV Filling Rate (EDV/s)	2.5 (2.1, 3.1)	2.9 (2.5, 3.4)	**0.04**

<u>Supplementary Table 6</u> – Expanded Diastology results for Study 1 and Study 2

Study Part 2

	Pre	Post	p
E/A (SR only)	0.81 (0.74,0.87)	0.84(0.80,1.01)	0.65
E/e medial	9.6 (8.8,11.6)	10.7 (8.4,13.0)	0.59
E/e lateral	6.7 (5.6,9.2)	6.8 (5.7,9.8)	0.51
Average E/E’	9.0 (7.1,9.8)	9.1 (6.7,10.6)	0.72
Normalised Peak LV Filling Rate (EDV/s)	3.4 (2.8,4.5)	3.3 (2.9,5.3)	0.46

Supplementary Figures

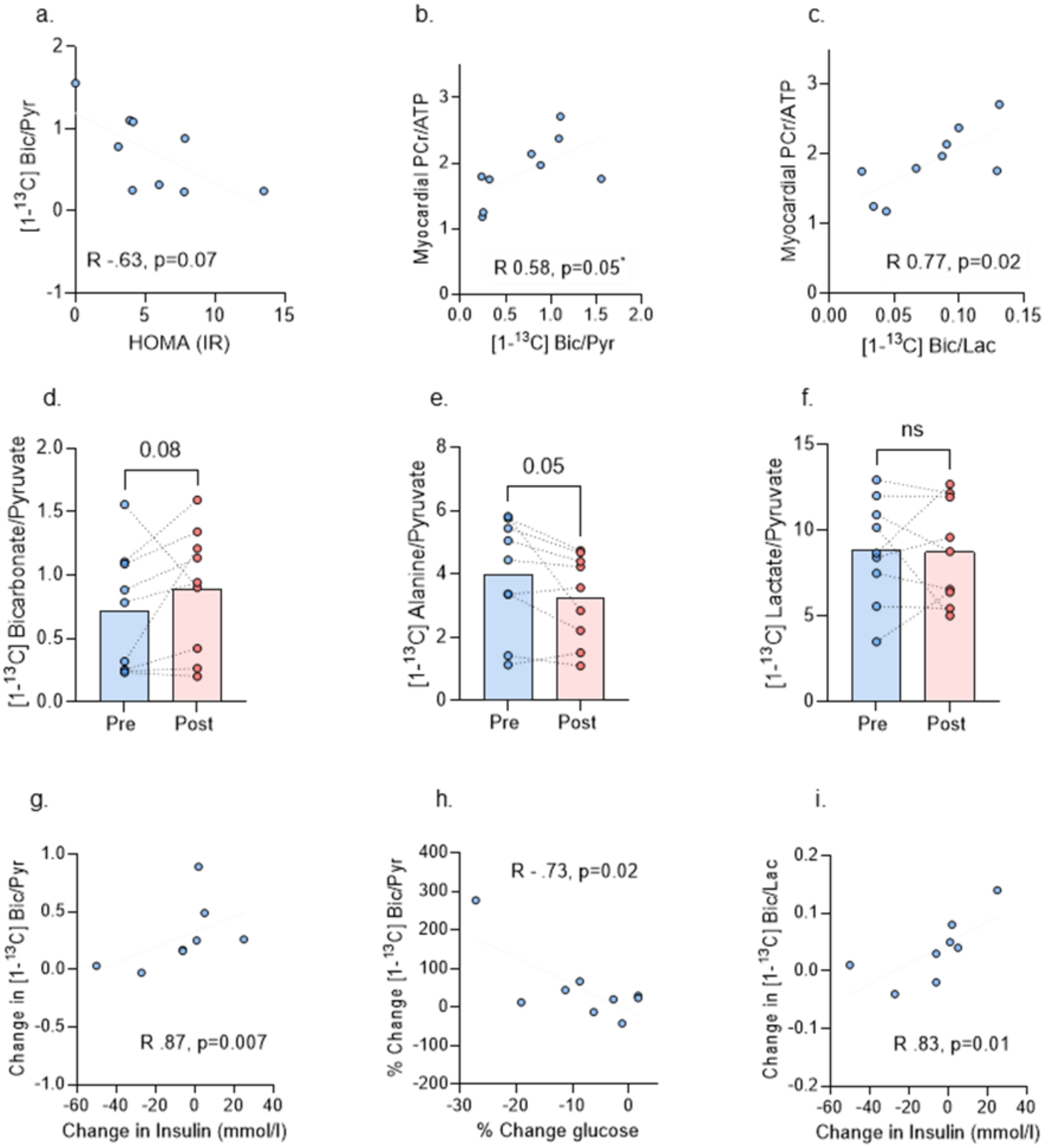

<u>Supplementary Figure S1</u> Hyperpolarized ^{13}C data for Study Part 1. Correlations between metabolic makers in Study Part 1. The relationship between hyperpolarized cardiac [1-^{13}C]-pyruvate magnetic resonance spectroscopy determined bicarbonate/pyruvate ratio before treatment and insulin resistance, **S1a** insulin resistance, **S1b** PCr/ATP, and 1c PCr/ATP and bicarbonate/lactate ratio. **S1d-f** show change in bicarbonate/pyruvate, alanine/pyruvate and lactate/pyruvate after ninerafaxstat. Change in pyruvate metabolism and change in glucose and insulin are shown in **S1g-h**, with change in bicarbonate/lactate ratio and change in insulin in **S1i**

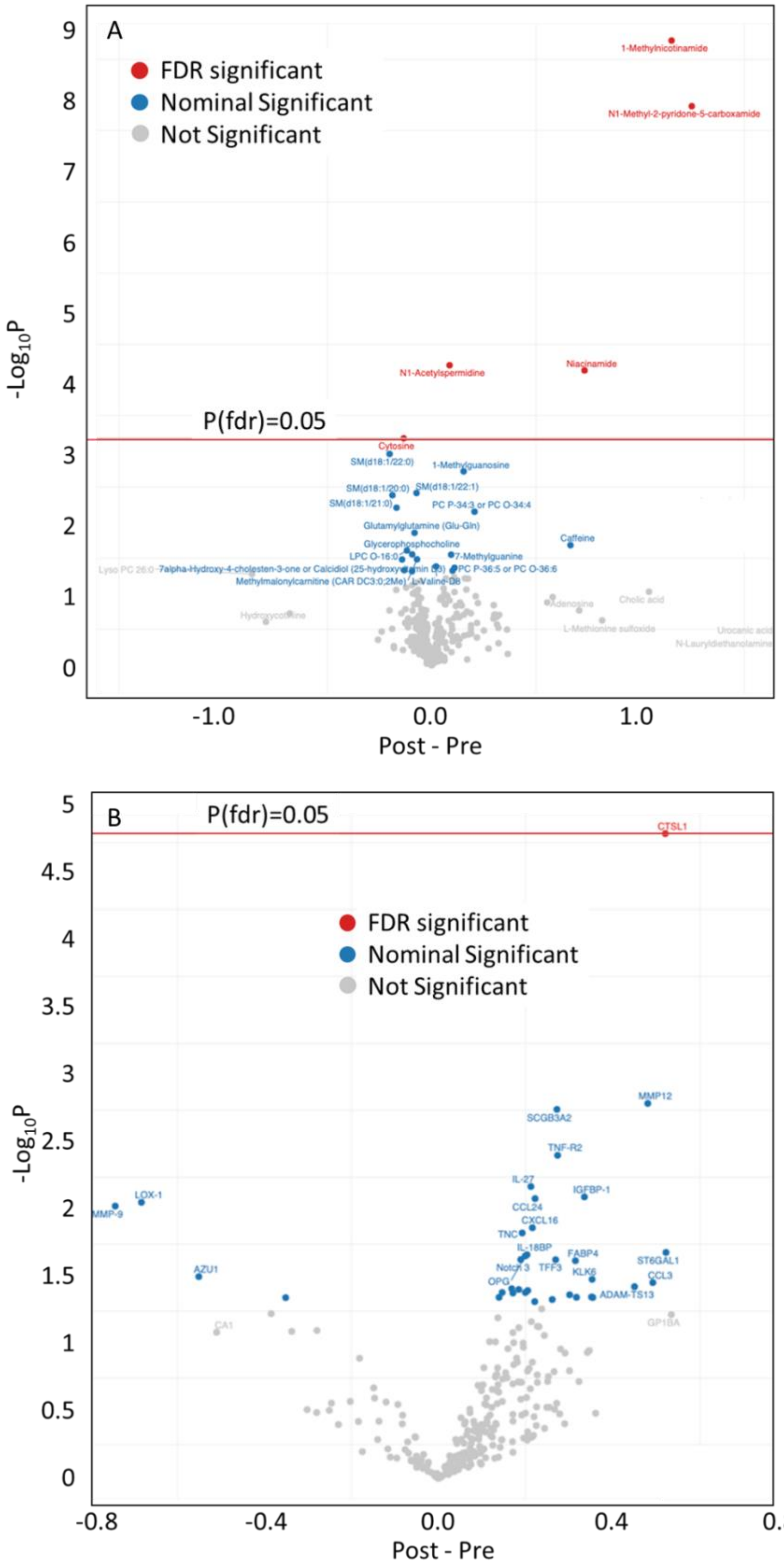

<u>Supplementary Figure 2</u> Proteomic and Metabolomic changes in Study Part 1; A Proteomic, and B Metabolomic changes.

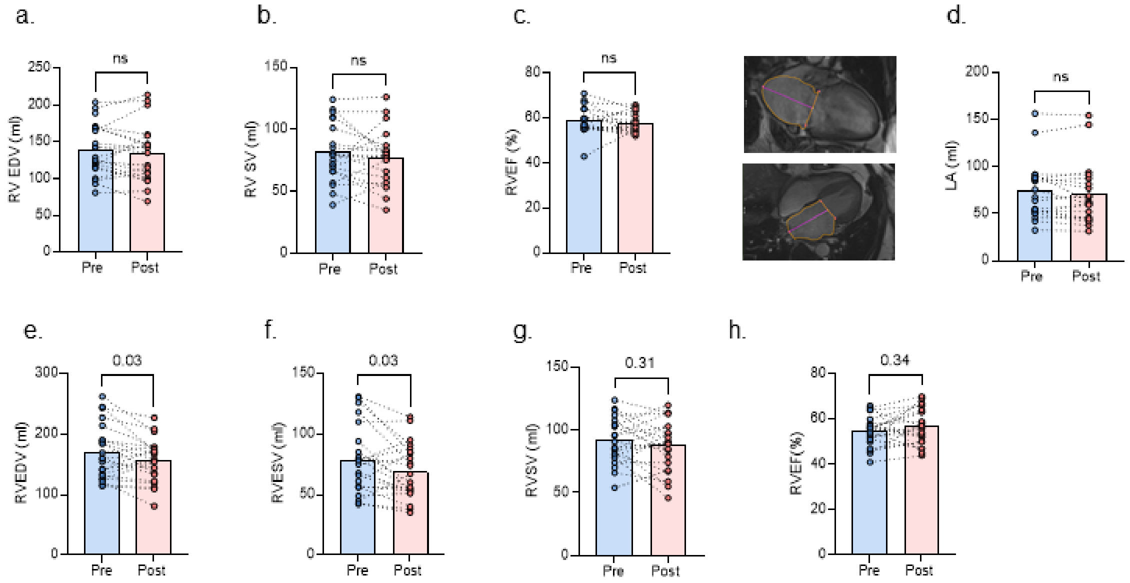

<u>Supplementary Figure S3</u> Right Ventricular and Left Atrial changes seen in Study Part 1 (**S3a-d**) and right ventricular (RV) changes in Study Part 2 (**S3e-h**)

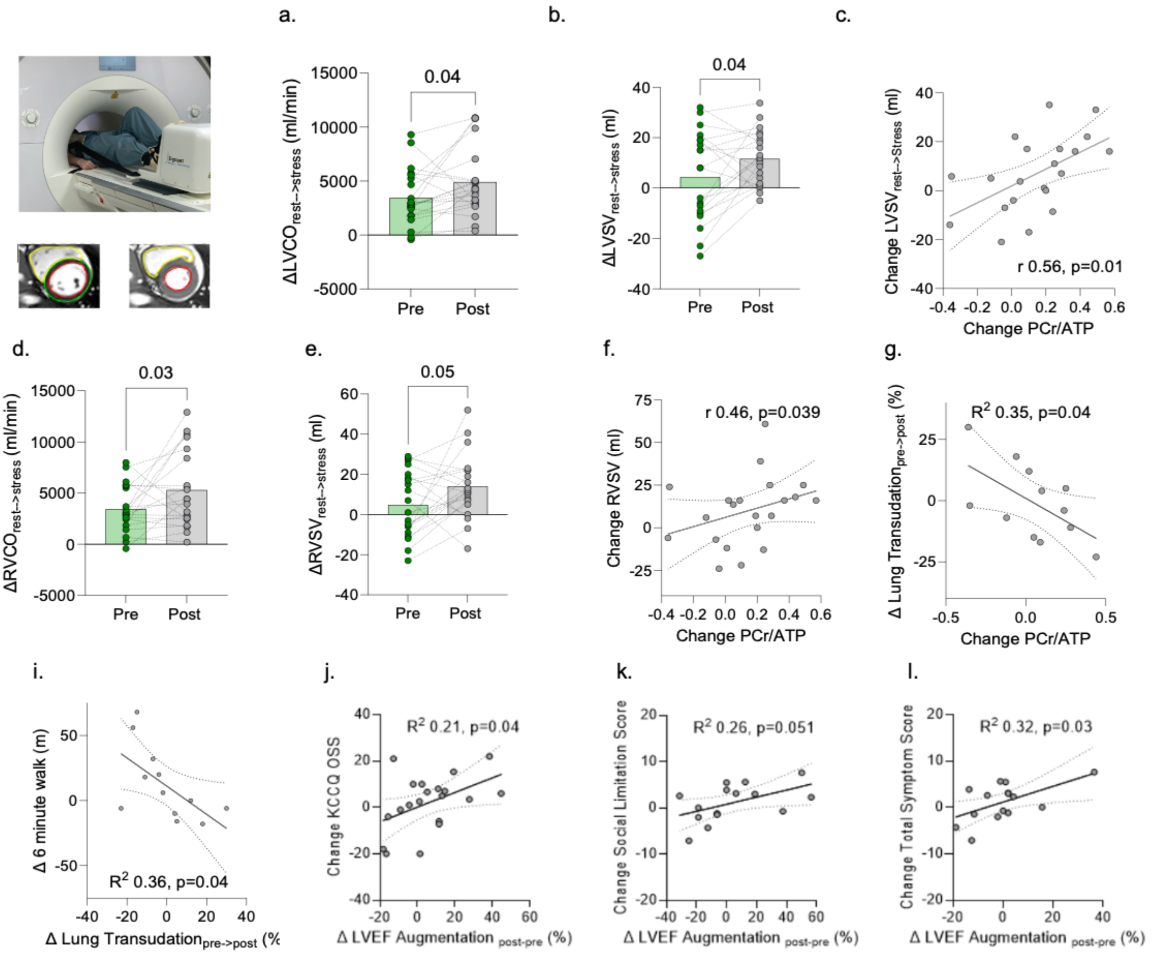

<u>Supplementary Figure S4</u>– Study Part 2 - The effect of ninerafaxstat on augmentation of cardiac output during fixed 30-watt (w) exercise in patients with cardiometabolic heart failure with preserved ejection fraction (HFpEF). **S4a** shows the greater increase in left ventricular cardiac output following treatment, with **S4b** showing left ventricular stroke volume. The relationship between stroke volume augmentation and change in PCr/ATP is shown in **S4c**. **S4d-e** show the corresponding right ventricular measures. **S4f-g** show the relationship between lung water transudation and change in PCr/ATP and 6-minute walk distance, **S4h-k** shows the relationship between change in left ventricular ejection fraction (LVEF) augmentation and patient reported outcomes

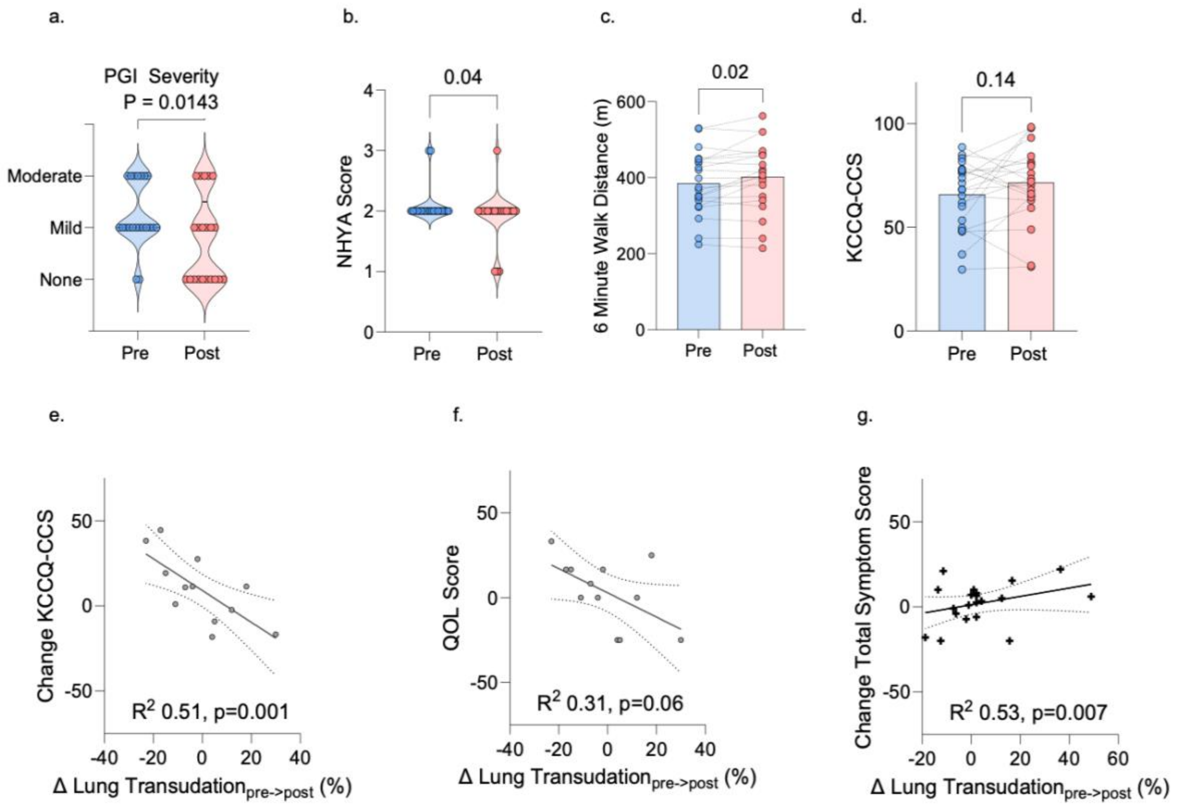

Supplementary Figure S5 **S5a** Patient Global Impression of Severity (PGI) score, **S5b** New York Heart Association (NHYA) class, **S4c** 6 minute walk distance, **S4d** Kansas City Cardiomyopathy Questionnaire Clinical Summary Score (KCCQ-CCS) pre and post intervention, **S4e-f** Correlations between change in KCCQ-CCS, quality of Life score (QOL) and Total Symptom score and lung water transudation following treatment with Ninerafaxstat in study part 2.

Extended Methods

Hyperpolarized ^{13}C-MRS

Sterile fluid pathways (SFPs) were assembled in a Grade A sterile environment containing 1.47 g [1-^{13}C]pyruvic acid (Sigma Aldrich, Gillingham, UK) and 15 mM AH111501 (Syncom, Groningen, Netherlands) as the electron paramagnetic agent (EPA). SFPs were loaded into a General Electric SpinLab system (GE Healthcare, Chicago, USA) which was used for the process of Dynamic Nuclear Polarization. Sufficient polarization levels were achieved after 2-3 hours. Dissolution was undertaken using 38.5 g of sterile water heated to 130°C under pressure, released through the pyruvate containing vial into a receiver vessel containing 17.7 g of trometamol buffer solution (600 mM NaOH, 333 mM Tris base, and 333 mg/L disodium EDTA [as the chelating agent], Royal Free Hospital, London, UK) and a further 19.5 g of sterile water. The EPA was removed by filtration prior to the receiver vessel, with the final product for injection drawn from the receiver vessel into a 50 ml injection syringe (Bayer, Indianola, USA) via a further 0.2 µm sterilization filter (Saint-Gobain, Gaithersburg, USA). Rigorous quality control (QC) of the final filtered sodium [1-^{13}C]pyruvate solution was undertaken prior to human injection. This consisted of both online measurements (pyruvate concentration, residual EPA concentration, temperature, polarization, volume) directly from the SpinLab inbuilt QC console, with further 'offline' pH measurement (RQflex 10, Merck, Darmstadt, Germany) and visual inspection of the product (for visible particulates and appearance) undertaken manually prior to release. Pathways were only released for human injection if the following criteria were met: pH 6.7-8.4, temperature 25.0-37.0°C, polarization ≥ 15%, [pyruvate] 220-280 mM, [EPA] ≤ 3.0 µM, appearance: clear, colourless solution with no visible particulate matter. Pathways not meeting these release criteria were rejected. Hyperpolarized [1-^{13}C]pyruvate solution was administered through an 18G venous cannula sited in the left antecubital fossa, at a dose of 0.4 ml/kg, followed by a 25 ml 0.9% normal saline flush. Injections were performed at a rate of 5 ml per second using a MEDRAD® power injector system (Bayer, Berlin, Germany).

^{13}C Spectral Analysis

Multi-coil data were recombined in MATLAB using the Whitened Singular Value Decomposition algorithm, with coil combination weights calculated for spectra with the highest SNR subsequently applied to the entire dataset. Spectra were background-subtracted prior to quantification with the AMARES algorithm, with appropriate prior knowledge. Total integrated metabolite-to-pyruvate ratios, known to linearly correlate with first-order chemical kinetic rate constants, were calculated from 60 seconds of data taken after the initial appearance of the pyruvate resonance in the spectrum.

^{31}P-MRS and Data Processing

All scans were performed on a Siemens 3T Tim Trio system (Erlangen, Germany). A Siemens Heart/Liver ^{31}P coil was used consisting of a large outer element (26 x 28 cm) which acts as ^{1}H transmit-receive and ^{31}P transmit, with a smaller loop/butterfly receive pair (12 x 15 cm loop and 23 x 12 cm butterfly) which receives ^{31}P signal. Subjects lay prone with their left ventricle positioned over the centre of the coil at the magnet iso-center. Proton localisers were used to position the subject correctly. Ten free induction decay inversion recovery (IR-FID) curves (1 ms hard inversion) with increasing inversion

delay (100 – 3000 ms) are acquired, along with locations of phenylphosphonic acid (PPA) fiducial and codliver-oil phantoms. Pilot images were taken to position the CSI matrix. Piloting was performed in vertical long axis (VLA), horizontal long axis (HLA) and short axis planes, where a stack of 20 slices was obtained. Fast low-angle shot (FLASH) images were used: slice thickness 10 mm, TR 7 ms, TE 3.37 ms, FOV 400 x 340 mm.

A 3-D acquisition-weighted chemical shift imaging (CSI) was used with an acquisition matrix size measuring 16 x 8 x 8 and a field of view of 240 x 240 x 200 mm resulting in an average voxel size of 11.25 ml. The grid was oriented to place voxels in the interventricular septum. Two saturation bands were placed over the skeletal muscle in the chest wall and one over the liver to minimise signal contamination. The acquisition was non-gated with TR around 910-1010 ms depending upon SAR. The acquisition delay was reduced to a minimum (TE* = 0.3 ms) using the ultra-short echo time (UTE-CSI) technique to maximise acquired signal and reduce first order phase effects (therefore reducing artefact). The optimised RF pulse (duration 2.4 ms) was centred between γ and α peaks (usually by subtracting 250 Hz from the observed phosphocreatine frequency) to ensure uniform excitation[63]. Exploitation of the Nuclear Overhauser effect (NOE) was used to increase the signal-to-noise ratio in acquired spectra: five pulses, length 2.5 ms, inter-pulse delay 80.5 ms, pulse voltage 222.5 V and average flip angle 150°.

<u>^{31}P Spectral Analysis</u>
The basal septal voxel was selected for analysis: this was the only user-dependent part of the process, the rest being fully automated. In-house Matlab software (OXSA [28]) determined the flip angle for the selected voxel by co-registering the short axis images with the spectral data. Flip angle variation due to coil loading effects was calculated using acquired inversion recovery data and the localizer containing the locations of cod liver oil capsules in fixed positions in the coil.
Pre-processing (baseline correction) was undertaken before fitting spectral peaks using the AMARES (advanced method of accurate, robust and efficient spectroscopic fitting) method. Peaks for phosphocreatine, α, β, γ - ATP, 2,3-diphosphoglycerate and phosphodiesters were fitted using prior knowledge of relative peak frequencies, J-coupling constants for ATP, relative peak amplitudes, relative phases and assumed Lorentzian line shapes along with acquisition parameters (central frequency, bandwidth, TR and calculated flip angles at various depths). Peak areas were corrected for RF partial saturation effects using the recorded excitation flip angle, T_1 values (PCr 3.8s, γ -ATP 2.4s, α-ATP 2.5s, β-ATP 2.7s, 2,3-DPG 1.39s, PDE 1.11s) and spectral overlap with the NADH peak. The value of the ATP peak was corrected for blood contamination by subtracting 11% of the DPG peak area[63]. PCr/ATP was calculated using the average of the three ATP peaks. The quality of spectral fit was assessed using the coefficient of variation in the measured PCr/ATP ratio, based on Cramer-Rao lower bounds (an indicator of signal to noise ratio in the sample) and standard error propagation formulae. Samples with a greater than 30% coefficient of variation were excluded.

<u>Metabolomic profiling</u>
Raw metabolomic data were processed using TraceFinder 3.1 (Thermo Fisher Scientific; Waltham, MA) and Progenesis QI (Nonlinear Dynamics; Newcastle upon Tyne, United Kingdom). LC-MS data were analyzed with Agilent Masshunter QQQ Quantitative

analysis software. Isotope-labeled internal standards were monitored in each sample to ensure adequate MS sensitivity for quality control. Peaks were manually reviewed in a blinded fashion to assess quality.

Proteomic profiling

The Proximity Extension Assay technology uses pairs of oligonucleotide-labeled antibody probes to bind to their target protein. If the two probes are brought in close proximity, the oligonucleotides hybridize in a pair-wise manner. The addition of a DNA polymerase results in proximity-dependent DNA polymerization, which creates a unique double-stranded DNA barcode for each specific antigen. Next-generation sequencing (Illumina NovaSeq) is then used to detect and quantify this DNA sequence. Data are quality-controlled and normalized using an internal extension control. The final assay readout is displayed as Normalized Protein eXpression (NPX) values, which are log2-transformed ratios of sample assay counts to extension control counts. All assay validation data are available on manufacturer's website (www.olinkexplore.com).
